# *Arabidopsis* pentatricopeptide repeat protein SOAR1 plays a critical role in abscisic acid signalling

**DOI:** 10.1093/jxb/eru293

**Published:** 2014-07-08

**Authors:** Chao Mei, Shang-Chuan Jiang, Yan-Fen Lu, Fu-Qing Wu, Yong-Tao Yu, Shan Liang, Xiu-Jing Feng, Sergi Portoles Comeras, Kai Lu, Zhen Wu, Xiao-Fang Wang, Da-Peng Zhang

**Affiliations:** MOE Systems Biology and Bioinformatics Laboratory, Center for Plant Biology, School of Life Sciences, Tsinghua University, Beijing 100084, China

**Keywords:** Abscisic acid signalling, *Arabidopsis thaliana*, Mg-chelatase H subunit, pentatricopeptide repeat (PPR) protein, post-germination growth, seed germination.

## Abstract

A dominant *s*uppressor *o*f the *ABAR* overexpressor, *soar1-1D*, from *CHLH/ABAR* [coding for Mg-chelatase H subunit/putative abscisic acid (ABA) receptor (ABAR)] overexpression lines was screened to explore the mechanism of the ABAR-mediated ABA signalling. The *SOAR1* gene encodes a pentatricopeptide repeat (PPR) protein which localizes to both the cytosol and nucleus. Down-regulation of *SOAR1* strongly enhances, but up-regulation of *SOAR1* almost completely impairs, ABA responses, revealing that SOAR1 is a critical, negative, regulator of ABA signalling. Further genetic evidence supports that SOAR1 functions downstream of ABAR and probably upstream of an ABA-responsive transcription factor ABI5. Changes in the *SOAR1* expression alter expression of a subset of ABA-responsive genes including *ABI5*. These findings provide important information to elucidate further the functional mechanism of PPR proteins and the complicated ABA signalling network.

## Summary

The authors identify a pentatricopeptide repeat (PPR) protein SOAR1 as a crucial player of ABA signalling, which localizes to both the cytosol and nucleus probably to regulate nuclear gene expression.

## Introduction

Pentatricopeptide repeat (PPR) proteins are a class of RNA-binding proteins characterized by the presence of a degenerate 35 amino acid repeat, the PPR motif, which is repeated in tandem 2–50 times. The PPR motifs form a helical structure and are considered to be RNA-binding motifs (Aubourg *et al.*, 2000; Small and Peeters, 2000; Lurin *et al.*, 2004). The first PPR gene was identified in *Saccharomyces cerevisiae* (Manthey and McKewen, 1995), and it is known that all sequenced eukaryotic genomes have been found to encode PPR proteins, though the numbers of PPR genes in both animal and fungal genomes are relatively small. The PPR domain protein family is particularly large in land plants. In the *Arabidopsis thaliana* genome, 450 putative PPR genes were identified, and >600 PPR genes have been predicted to occur in the rice genome (Small and Peeters, 2000; Lurin *et al.*, 2004; Rivals *et al.*, 2006; Schmitz-Linneweber and Small, 2008).

It has been known that PPR proteins are mostly targeted to mitochondria and/or chloroplasts in plants, and they are involved in many aspects of RNA processing in these two organelles, such as RNA splicing, editing, 5′ and 3′ end processing, stability and cleavage, and translation (Meierhoff *et al.*, 2003; Williams and Barkan, 2003; Lurin *et al.*, 2004). The mitochondrial/chloroplast PPR proteins play diverse and crucial roles in plant developmental processes and responses to environmental stresses (Small and Peeters, 2000; Lurin *et al.*, 2004; Oguchi *et al.*, 2004; Tzafrir *et al.*, 2004; Cushing *et al.*, 2005; Ding *et al.*, 2006; Wang *et al.*, 2006; Gutierrez-Marcos *et al*., 2007; Koussevitzky *et al.*, 2007; Chi *et al.*, 2008; Fujii and Small, 2011; Hu *et al.*, 2012; Nakamura *et al.*, 2012). Few PPR proteins, however, have been found to localize to cellular compartments other than mitochondria and chloroplasts. To the authors’ knowledge, thus far, two PPR proteins have been identified as localizing to the nucleus, of which one PPR protein was found only in the nucleus and another in both the mitochondrion and the nucleus, and they regulate embryogenesis probably by modulating nuclear gene transcription and RNA processing (Ding *et al.*, 2006; Hammani *et al.*, 2011).

The phytohormone abscisic acid (ABA) regulates many developmental processes and plant adaptation to adverse conditions (reviewed in Finkelstein *et al.*, 2002; Adie *et al.*, 2007; Cutler *et al.*, 2010). Numerous ABA signalling components, including receptors or candidate receptors for ABA, have been identified (Finkelstein *et al.*, 2002, Gao *et al.*, 2007; Johnston *et al.*, 2007; Liu *et al*., 2007*a*, *b*; Guo *et al.*, 2008; Ma *et al.*, 2009; Pandey *et al.*, 2009; Park *et al.*, 2009; Santiago *et al.*, 2009; Cutler *et al.*, 2010). The START-domain family proteins PYR/PYL/RCAR are the best characterized cytosolic ABA receptors, which mediate a core ABA signalling pathway involving the downstream components such as the type 2C protein phosphatases (PP2Cs), SNF1-related protein kinase 2s (SnRK2s), and a clade of bZIP-domain transcription factors (Fujii *et al.*, 2009; Ma *et al.*, 2009; Park *et al.*, 2009; Santiago *et al.*, 2009; Cutler *et al.*, 2010). In the highly complex ABA signalling network, five *Arabidopsis* PPR proteins, PPR4 (Zsigmond *et al.*, 2008), ABO5 (Liu *et al.*, 2010), PGN (Laluk *et al.*, 2011), SLG1 (Yuan and Liu, 2012), and AHG11 (Murayama *et al.*, 2012), have been identified to play an important role. All five PPR proteins were found to localize to mitochondria and probably regulate reactive oxygen species (ROS) production in this organelle to be involved in ABA signalling (Zsigmond *et al.*, 2008; Liu *et al.*, 2010; Laluk *et al.*, 2011; Murayama *et al.*, 2012; Yuan and Liu, 2012).

It was previously reported that the chloroplast magnesium-protoporphyrin IX chelatase large subunit [Mg-chelatase H subunit CHLH/putative ABA receptor (ABAR)] functions as a candidate receptor for ABA in *Arabidopsis* (Shen *et al.*, 2006; Wu *et al.*, 2009; Shang *et al.*, 2010; Du *et al.*, 2012; Liu *et al.*, 2012; Yan *et al.*, 2013; Zhang *et al*., 2013, 2014). Although it is still controversial whether CHLH/ABAR binds ABA (Shen *et al.*, 2006; Muller and Hansson, 2009; Wu *et al.*, 2009; Tsuzuki *et al.*, 2011; Wang *et al.*, 2011; Du *et al.*, 2012), it has been well supported that CHLH/ABAR functions positively in ABA signalling by regulating a complicated pathway, in which the chloroplast protein cochaperonin CPN20 and cytosolic–nuclear protein WRKY18/40/60 transcription repressors are involved (Shen *et al.*, 2006; Wu *et al.*, 2009; Shang *et al.*, 2010; Du *et al.*, 2012; Liu *et al.*, 2012; Yan *et al.*, 2013; Zhang *et al*., 2013, 2014). There are four *abar* mutant alleles in *Arabidopsis*, *abar-2*, *abar-3*, *cch*, and *rtl1*, which all show altered ABA responses (Shen *et al.*, 2006; Wu *et al.*, 2009; Tsuzuki *et al*., 2011, 2013; Du *et al.*, 2012). It was shown that CHLH/ABAR also regulates guard cell signalling in response to ABA in tobacco (*Nicotiana benthamiana*) leaves (Du *et al.*, 2012). Other independent groups demonstrated that CHLH/ABAR mediates ABA signalling in guard cells in both *Arabidopsis* (Legnaioli *et al.*, 2009; Tsuzuki *et al*., 2011, 2013) and peach (*Prunus persica*) leaves (Jia *et al.*, 2011*a*). Tsuzuki and co-workers (2013) recently showed that CHLH/ABAR mediates ABA inhibition of blue light (BL)-induced phosphorylation of H^+^-ATPase in *Arabidopsis* guard cells, suggesting that CHLH/ABAR regulates not only ABA-induced stomatal closure but also ABA inhibition of BL-mediated stomatal opening. Interestingly, it has been demonstrated that CHLH/ABAR mediates ABA signalling in fruit ripening of both peach (Jia *et al.*, 2011*a*) and strawberry (*Fragaria ananassa*) (Jia *et al.*, 2011*b*). These data demonstrate that CHLH/ABAR is an essential ABA signalling regulator in plant cells.

To explore further the mechanism of the CHLH/ABAR-mediated ABA signalling, a suppressor of the *ABAR* overexpressor (named *soar1-1D*), in which the expression levels of both the *CHLH*/*ABAR* gene and the *SOAR1* gene encoding a PPR-motif protein, are up-regulated, was screened. It was shown that SOAR1 localizes to both the cytosol and nucleus, and functions as a critical, negative, regulator of the ABA signalling pathway in seed germination and seedling growth. Genetic evidence revealed that SOAR1 acts downstream of ABAR and probably upstream of a nuclear ABA-responsive bZIP transcription factor ABA-INSENSITIVE5 (ABI5). These findings provide important information to elucidate further the functional mechanism of PPR proteins and the highly complicated ABA signalling network.

## Materials and methods

### Plant materials and growth conditions

*ABAR* overexpression lines were generated by introducing an *ABAR* gene (At5g13630) fragment (encoding a truncated ABAR with amino acid residues 631–999) into *A. thaliana* ecotype Columbia-0 (Col) plants as a green fluorescent protein (GFP) fusion protein. It was previously shown that the N-terminally truncated ABAR tagged with GFP functions similarly to full-length ABAR in transgenic plants, leading to ABA hypersensitivity in the major ABA responses (Wu *et al.*, 2009). Therefore, the truncated *ABAR* overexpression lines were used as *ABAR* overexpressors. The cDNA isolation and transgenic manipulation were as previously described (Wu *et al.*, 2009). From the population of the *ABAR* overexpression transgenic lines, the lines with ABA-insensitive or wild-type phenotypes in seed germination and post-germination growth were screened, which gave candidate suppressors of the *ABAR* overexpressor (named *soar* mutant) lines. The *soar1-1D* mutant was identified from these candidate *soar* mutant lines.

The *soar1-2* and *soar1-3* (stock nos FLAG_546D07and FLAG_500B04, respectively, with the Col ecotype as background) were obtained from Versailles Genetics and Plant Breeding Laboratory, *Arabidopsis thaliana* Resource Centre (INRA; http://dbsgap.versailles.inra.fr/portail/). The seed of the *abi5-1* (stock no. CS8105, with the Wassilewskija ecotype as background; locus of the *ABI5* gene, At2g36270) mutant was obtained from the Arabidopsis Biological Resource Center (ABRC; http://abrc.osu.edu/), and the background of the *abi5-1* mutant was changed to the Col ecotype by crossing as described previously (Shang *et al.*, 2010). The *soar1-2 abi5-1* double mutant was created by crossing. The seeds of *abi1-3* (stock no. SALK_076309) and *abi2-2* (stock no. SALK_015166) mutants were also obtained from the ABRC, and the mutants are T-DNA insertion knockout alleles in the *ABI1* (At4g26080) and *ABI2* (At5g57050) genes, respectively. The background of both mutants is the Col ecotype. The *abi1-3 abi2-2* double mutant is a generous gift from Y. Guo’s laboratory (College of Biological Sciences, China Agricultural University). All the primers for identification of the above-mentioned mutants are listed in Supplementary Table S1 available at *JXB* online.

The *A. thaliana* ecotype Col was used to generate transgenic plants. To generate the *SOAR1* (At5g11310) overexpression lines, the full-length *SOAR1* cDNA, amplified by PCR with the primers listed in Supplementary Table S1 at *JXB* online, was cloned into the binary vector pCAMBIA1300 (http://www.cambia.org), which contains the *Cauliflower mosaic virus* (CaMV) *35S* promoter and the C-terminal GFP flag. Also, an *ABAR* and *SOAR1* double gene overexpression line was created by crossing an *ABAR* overexpressor with a *SOAR1* overexpressor (OE1) to test genetic interaction of these two genes. A previously generated *ABI2* overexpression line (Sun *et al.*, 2011; the ABI2-2 line harbouring GFP-tagged full-length *ABI2* under the Col background) was used as a control in phenotypic analysis of the *SOAR1* overexpression lines, which showed strong ABA-insensitive phenotypes (Sun *et al.*, 2011) and was renamed ABI2-OE in the present study. To generate the transgenic complementation lines of the *soar1-2* and *soar1-3* mutants, the native promoter, isolated by PCR with the primers listed in Supplementary Table S1 was used, to replace the CaMV *35S* promoter in the above-mentioned construct to create the native promoter-driven *SOAR1* construct. These constructs were introduced, respectively, into *Agrobacterium tumefaciens* strain GV3101 and transformed into the wild-type plants (for the *SOAR1*-overexpressiong lines) or *soar1* mutants (for the complementation lines of the *soar1-2* and *soar1-3* mutants) by the floral dip infiltration method (Clough and Bent, 1998). The homozygous T_3_ seeds of the transgenic plants were used for analysis.

Plants were grown on Murashige and Skoog (MS) medium (Murashige and Skoog, 1962) containing 3% (w/v) sucrose and 0.8% agar or in compost soil under a 16h photoperiod in a growth chamber at ~20 °C. Plants were grown under a 12h photoperiod at ~20 °C for protoplast preparation.

### SOAR1 promoter–GUS transformation assay

The promoter of *SOAR1* (*pSOAR1*) was amplified by PCR using forward primer 5ʹ-AACTGCAGTTCCGACAAACATAAAATGG TA-3ʹ and reverse primer 5ʹ-CGGGATCCTCCGCCGAGAAAATT AGGACA-3ʹ. The PCR product was digested and cloned into the pCAMBIA1391 vector. The construct *pSOAR1*-*GUS* (*β-glucuronidase*) was transformed into *Arabidopsis* Col plants by floral infiltration. Histochemical staining was performed, as described previously (Jefferson *et al.*, 1987), by soaking whole plants or tissues in a solution consisting of 1mM X-gluc, 100mM sodium phosphate buffer (pH 7.0), 0.05mM K_3_Fe(CN)_6_, 0.05mM K_4_Fe(CN)_6_, 2mM EDTA, and 0.1% (v/v) Triton X-100 at 37 °C for 5–8h. After GUS staining, chlorophyll was cleared from the tissues with a mixture of 30% acetic acid and 70% ethanol, and then the samples were investigated under a stereomicroscope (Olympus).

### Quantitative real-time PCR and TAIL-PCR

Quantitative real-time PCR for mRNA expression levels of various genes (see Supplementary Table S1 at *JXB* online for the gene-specific primers) was performed as previously described (Shang *et al.*, 2010) essentially according to the instructions provided for the Bio-Rad Real-Time System CFX96TM C1000 thermal cycler (Bio-Rad, Hercules, CA, USA). The T-DNA flanking sequence in the *soar1-1* dominant mutant was determined by TAIL-PCR (thermal asymmetric interlaced PCR) with pCAMBIA1300-specific left border and random primers that are listed in Supplementary Table S1. The reaction program for rounds was described previously (Liu *et al.*, 1995).

Seeds were stratificated for 3 d at 4 °C, incubated on filter paper imbibed with ABA-free or ABA-containing solution for 24h in a light growth chamber at 20 °C, and collected for RNA extraction. Total RNA was isolated from these germinating seeds with the RNasy plant mini kit (Qiagen) supplemented with an on-column DNA digestion (Qiagen RNase-Free DNase set) according to the manufacturer’s instructions, and then the RNA sample was reverse-transcribed with the Superscript II RT kit (Invitrogen) in a 25ml volume at 42 °C for 1h. Amplification of ACTIN2/8 genes was used as an internal control. The cDNA was amplified using SYBR Premix Ex Taq (TaKaRa) with a DNA Engine Opticon 2 thermal cycler in a 10ml volume. The Ct (threshold cycle), defined as the PCR cycle at which a statistically significant increase of reporter fluorescence was detected, was used as a measure for the starting copy numbers of the target gene. Relative quantitation of the target gene expression level was performed using the comparative Ct method.

### Arabidopsis *protoplast and onion epidermis transformation*

Transient transgenic manipulation in both *Arabidopsis* protoplasts and onion epidermis was used to assay the subcellular localization of the SOAR1 protein essentially as described previously (Shang *et al.*, 2010). The full-length *SOAR1* and a fragment of 106–1809bp downstream of the transcription start site of *SOAR1* (*SOAR1* ^*106–1809*^, which encodes a truncated SOAR1 from amino acid residue 36 to 603 with the N-terminal 35 amino acid residues deleted) were amplified by PCR, and the products were cloned into the p-EASY-T1 vector (Transgen, Beijing, China) for sequencing, and then fused with *GFP* and inserted into the pROK219 vector, driven by the CaMV *35S* promoter. The positive control cytosolic–nuclear marker *PYR1* (At4g17870; Park *et al.*, 2009) and nuclear marker *FBI1* (At1g02340; Lee *et al.*, 2002) were used as described previously (Zhao *et al.*, 2011), and their cDNAs were amplified and fused with *mCherry* (Shaner *et al.*, 2004) in-frame into the pROK219 vector, driven by the CaMV *35S* promoter, respectively. The primers for cloning the full-length *SOAR1*, *SOAR1* ^*106–1809*^, *PYR1*, and *FBI1* are listed in Supplementary Table S1 at *JXB* online. Protoplasts were transiently transformed using the polyethylene glycol-mediated transformation protocol (Yoo *et al.*, 2007). The onion epidermal cells were transformed by particle bombardment-mediated transformation with the biolistic PDS-1000/HE gene gun system (Bio-Rad). Bombarded samples were cultured at 26 °C for 16h, and then observed with a confocal laser scanning microscope (ZEISS, Oberkochen, Germany).

### Isolation of cytosolic and nuclear fractions

The cytosolic and nuclear fractions were isolated essentially according to the protocol described previously (Shang *et al.*, 2010). Ten-day-old *Arabidopsis* seedlings were ground to fine powder using liquid nitrogen and pre-chilled using a mortar and pestle. Cytosolic protein isolation buffer is composed of 10mM HEPES, pH 8.0, 250mM sucrose, 0.5% (v/v) Triton X-100, 1mM EDTA, 5mM MgCl_2_, 50mM NaCl, 1mM phenylmethylsulphonyl fluoride (PMSF), and 1× Roche Cocktail (protease inhibitor cocktail). The buffer was added at 1ml g^–1^ to powder to generate the homogenate. After centrifuging at 10 000 *g* for 15min, the supernatant was mixed with 2× SDS sample buffer and denatured for 10min in boiling water. The isolated cytosolic fraction was examined by immunodetecting the presence of the nuclear marker histone H3 with anti-histone H3 antibody (Sigma-Aldrich) to verify that the cytosolic fraction was not contaminated by the nuclear fraction. The nuclear fraction was isolated according to the protocol of Cold Spring Harbor Laboratory as described on its website, and examined by immunodetecting the presence of the cytosolic marker PEPC (phosphoenolpyruvate carboxylase) with anti-PEPC antibody (Agrisera) to ensure that the nuclear fraction was not contaminated by the cytosolic fraction.

### Antiserum production, protein extraction, and immunoblotting

The antisera against ABAR and SOAR1 were produced and tested for specificity essentially with the same procedures as described previously (Wu *et al.*, 2009; Shang *et al.*, 2010). A truncated SOAR1 (303 amino acid residues from 299 to 602) was used as antigen for production of the anti-SOAR1 serum. The primers for cloning the cDNA fragment to produce the truncated SOAR1 in *Escherichia coli* are listed in Supplementary Table S1 at *JXB* online. The produced anti-SOAR1 serum was tested and shown to be specific for SOAR1 protein (Supplementary Fig. S3). The extraction of the *Arabidopsis* total protein, SDS–PAGE, and immunoblotting were done essentially according to previously described procedures (Wu *et al.*, 2009; Shang *et al.*, 2010).

Ten-day-old *Arabidopsis* seedlings were harvested, ground in liquid nitrogen, then transferred into an Eppendorf tube containing ice-cold extraction buffer composed of 50mM TRIS-HCl, pH 7.5, 150mM NaCl, 1mM EDTA, 0.1% Triton X-100, 10% glycerol, and 1× protease inhibitor cocktail (Roche). The sample was extracted for 15min in ice, and centrifuged three times for 10min each at 16 000 *g*; the supernatant was transferred to a new Eppendorf tube and centrifuged again at 12 000 *g* for 20min, and then the concentration of the supernatant was detected by Coomassie Brilliant Blue G-250 (Amresco). The samples were either kept at 0 °C for immediate use or frozen and stored at –80 °C until use.

For the immunoblotting assays, proteins were separated by SDS–PAGE on 10% polyacrylamide gels, and transferred to nitrocellulose membranes (0.45 μm; Amersham Life Science) in a medium consisting of 25mM TRIS-HCl (pH 8.3), 192mM glycine, and 20% (v/v) methanol. After rinsing in TRIS-buffered saline (TBS) containing 10mM TRIS-HCl (pH 7.5) and 150mM NaCl, the blotted membranes were pre-incubated for 3h in a blocking buffer containing 3% (w/v) bovine serum albumin dissolved in TBS supplemented by 0.05% (v/v) Tween-20 (TBST1) and then incubated with gentle shaking for 2h at room temperature with appropriate antibodies. The anti-GFP serum (mouse, YTHX Biotechnology Beijing Limited Company, http://www.ythxbio.com/) and anti-actin serum (rabbit) were diluted ~1:3000, and the anti-SOAR1 serum (rabbit) was diluted ~1:2000 in the blocking buffer. Following extensive washes by TBST1, the membranes were incubated with goat anti-rabbit (or anti-mouse for GFP immunoblot) IgG (Cell Signaling Technology, http://www.cellsignal.com/) conjugated with alkaline phosphatase (diluted ~1:1000 in TBST1) at room temperature for 1h and then washed with TBST2 [50mM TRIS-HCl, pH 7.5, 150mM NaCl, and 0.1% (v/v) Tween-20] and TBS. The locations of antigenic proteins were visualized by incubating the membranes with nitroblue tetrazolium and 5-bromo-4-chloro-3-indolyl phosphate.

### Nucleus and mitochondrion staining in *Arabidopsis* roots

The roots of the 7-day-old OE1 seedlings were sampled and stained for 30min in 1 μg ml^–1^ 4′,6-diamidino-2-phenylindole (DAPI; Sigma; a nuclear marker), or 15min in 0.250 μM Mito Tracker Red CMXRos (Invitrogen). DAPI was dissolved in ddH_2_O directly. The Mito Tracker Red CMXRos (a mitochondrion marker) stock solution (250 μM) was dissolved in dimethylsulphoxide, then diluted 1000 times with ddH_2_O or 10mM phosphate-buffered saline (PBS) (pH 7.4, 10mM Na_2_HPO_4_, 10mM NaH_2_PO_4_, 8.5g l^–1^ NaCl) when used for staining. After the staining process, the samples were rinsed several times with ddH_2_O (for DAPI staining) or 10mM PBS (for the Mito Tracker Red staining). Samples were examined with a Leica TCS SP5 confocal microscope under a 63.0×1.40 oil immersion objective.

### Phenotypic analysis

Phenotypic analysis was carried out essentially as previously described (Shen *et al.*, 2006; Wu *et al.*, 2009; Shang *et al.*, 2010). Seeds were harvested and stored at room temperature for ~3–6 months before being used in the experiments. To assay germination and post-germination growth, the MS medium (Sigma-Aldrich, St Louis, MO, USA; full-strength MS) contained 3% (w/v) sucrose and 0.8% (w/v) agar, pH 5.8–6.0, and was supplemented or not with different concentrations of (±)-ABA. The seeds were sown and stratified in the MS medium at 4 °C for 3 d, and then they were placed at 20 °C under light conditions. Germination (emergence of radicals) was scored at the indicated times. Seedling growth was assessed by directly sowing the seeds in ABA-containing MS medium to investigate the response of seedling growth to ABA after germination. Another method was used to assay seedling growth in response to ABA: seeds were germinated after stratification on common MS medium and transferred to MS medium supplemented with different concentrations of (±)-ABA. The time for transfer was 48h or 4 d (as indicated) after stratification. Seedling growth was investigated at the indicated times after the transfer.

## Results

### *Down-regulation of* SOAR1 *increases, and up-regulation of* SOAR1 *abolishes, ABA sensitivity in seed germination and post-germination growth*

**Fig. 1.**
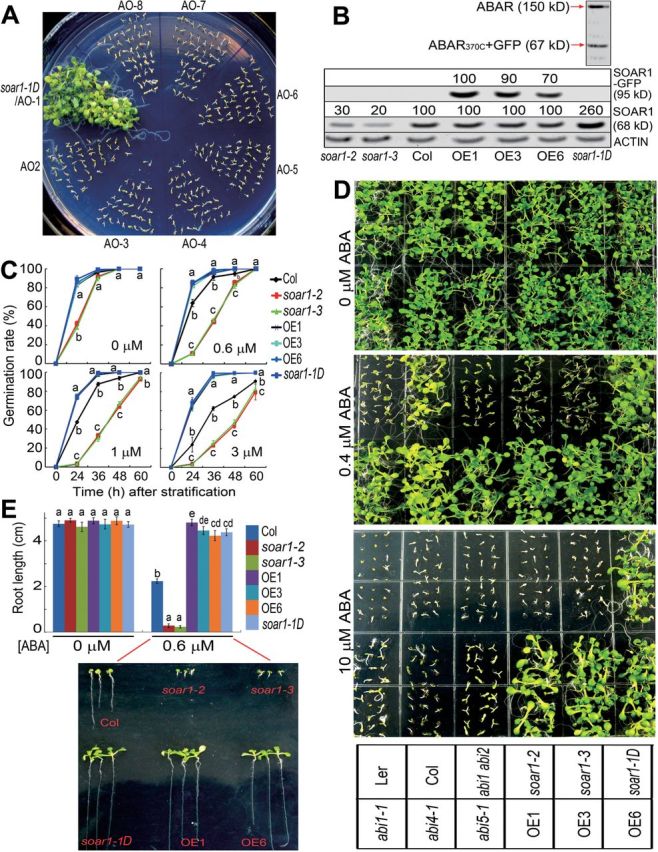
SOAR1 negatively regulates ABA signalling. (A) Screen of the *soar1-1D* mutant. AO1–AO8 indicate the *ABAR* overexpression lines 1–8. The seeds were directly planted in 1 μM ABA-containing MS medium, and seedling growth was investigated 10 d after stratification. (B) Immunoblotting assays for the SOAR1–GFP fusion (95kDa) and SOAR1 protein (68kDa) levels in the 10-day-old seedlings of the *soar1-1D*, *soar1-2*, and *soar1-3* mutants, and *SOAR1* overexpression lines (OE1, OE3, and OE6). The SOAR1–GFP fusion and SOAR1 protein amounts were evaluated by scanning the protein bands, and relative band intensities, normalized relative to the band intensity (as 100%) from the sample of the OE1 (for the SOAR1–GFP fusion) or the wild-type Col plants (for the SOAR1 protein), are indicated by numbers above the bands. Actin was used as a loading control. In the *soar1-1D* mutant, the expression of the truncated ABAR (ABAR_370C_) tagged by GFP (67kDa), introduced by transgenic manipulation, was tested, and the 150kDa wild-type ABAR was also detected. (C) Seed germination: germination rates of the wild-type Col, *soar1-1D*, *soar1-2*, and *soar1-3* mutants, and three *SOAR1* overexpression lines (OE1, OE3, and OE6) were recorded on ABA-free (0 μM) and ABA-containing (0.6, 1, or 3 μM) MS medium from 24h to 60h after stratification. (D) Early seedling growth: seeds from the wild types Col and Ler, the *abi1-1* dominant mutant, the *abi1-3 abi2-2* double-knockout mutant, *abi4-1* and *abi5-1* mutants, and the different genotypes as described in (C) were directly planted in the MS medium supplemented with 0 (top), 0.5 (middle), or 10 μM (±)ABA (bottom), and the growth was investigated 10 d after stratification. (E) Statistical values of the early seedling growth described in (D) from the wild-type Col, *soar1-1D*, *soar1-2*, and *soar1-3* mutants, and three *SOAR1* overexpression lines (OE1, OE3, and OE6) in the MS medium supplemented with 0 and 0.6 μM (±)ABA. The bottom panel shows the pictures of early seedling growth of these genotypes. Each value in (C) and (E) is the mean ±SE of five biological determinations, and different letters indicate significant differences at *P*<0.05 (Duncan’s multiple range test) when comparing the germination rates among different genotypes at the same time point after stratification (C) or comparing the root lengths among different genotypes in the ABA-free and 0.6 μM ABA-containing medium (E).

The *ABAR* overexpression lines show ABA-hypersensitive phenotypes (Shen *et al.*, 2006; Wu *et al.*, 2009; see also the Materials and methods). From the *ABAR* overexpression lines (see the Materials and methods), which were identified by PCR analysis, a putative *soar1* mutant named *soar1-1D* was screened, which showed an ABA-insensitive phenotype (Fig. 1A). PCR analysis showed that the construct for overexpressing the *ABAR* gene was inserted into the promoter of the *SOAR1* gene in the *soar1-1D* mutant (Supplementary Fig. S1 at *JXB* online). The *SOAR1* gene (At5g11310) encodes a PPR protein with tandem arrays of 10 predicted PPR motifs (Supplementary Fig. S2). The SOAR1 protein appears not to be a P-type member of the PPRs as it has a C-terminal extension (Supplementary Fig. S2) which is not related to other domains of PPRs according to its sequence. The GFP-tagged, functional, truncated ABAR was detected by immunoblot analysis in this *soar1-1D* mutant (Fig. 1B). To characterize the mutant further, the antibody against a truncated SOAR1 protein (303 amino acid residues from 299 to 602, see the Materials and methods) was generated, which was shown to be specific for SOAR1 (Supplementary Fig. S3). Immunoblot assays by using this anti-SOAR1 serum showed that the amount of SOAR1 protein in the *soar1-1D* mutant was enhanced >2-fold compared with the wild-type plants (Fig. 1B), which results from this ‘T-DNA’ (construct for overexpressing the *ABAR* gene) insertion (Supplementary Fig. S1). The *soar1-1D* is a dominant allele, of which both the homozygous and heterozygous progeny showed strong ABA-insensitive phenotypes in ABA-induced seed germination inhibition and post-germination growth arrest (Fig. 1A–E). Further, T-DNA insertion mutant alleles, *soar1-2* and *soar1-3*, which down-regulate the *SOAR1* expression level were obtained (Fig. 1B; Supplementary Fig. S1). A null allele of the *SOAR1* gene was not isolated, probably because the loss-of-function *soar1* mutant is lethal. It was observed that, in contrast to the *soar1-1D* mutant, the *soar1-2* and *soar1-3* mutants showed ABA-hypersensitive phenotypes in ABA-induced seed germination inhibition and post-germination growth arrest (Fig. 1C–E; Supplementary Fig. S4A–E). The seeds of the *soar1-2* and *soar1-3* mutants germinated more slowly than the wild-type seeds in the exogenous ABA-free medium, suggesting that these mutant seeds may be overly sensitive to the endogenous ABA at a low, physiological level (Fig. 1C; Supplementary Fig. S4A available at *JXB* online). The intensity of the ABA-hypersensitive phenotypes of the *soar1-2* and *soar1-3* mutants was similar to, or stronger than, that of the well-characterized *abi1 abi2* double-knockout mutant.

**Fig. 2.**
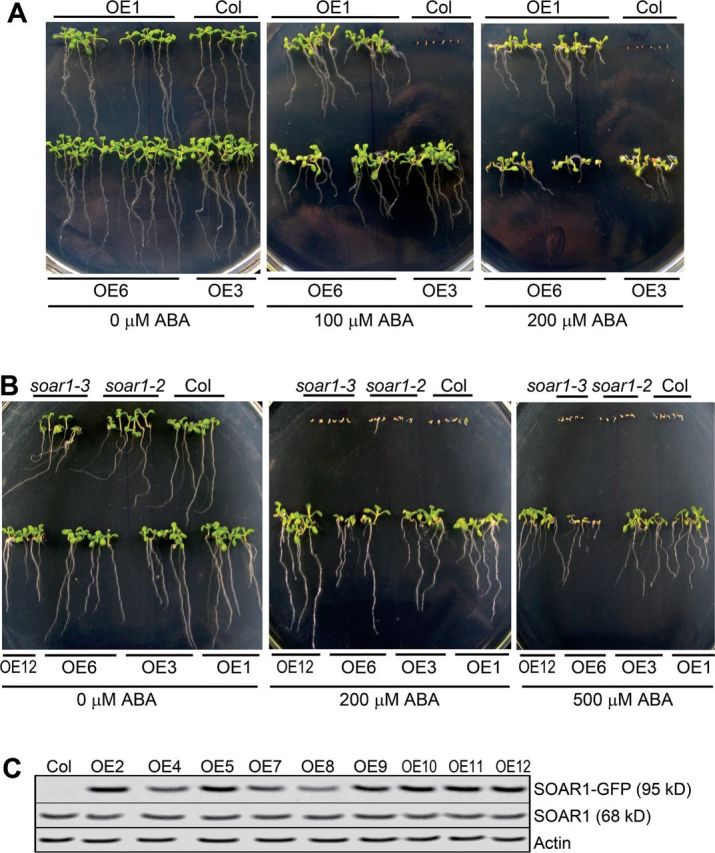
Overexpression of *SOAR1* essentially abolishes ABA responses of seeds and young seedlings. (A) Early seedling growth: seeds from the wild-type Col and three *SOAR1* overexpression lines (OE1, OE3, and OE6) were directly planted in MS medium supplemented with 0 (left), 100 (middle), or 200 μM (±)ABA (right), and the growth was investigated 2 weeks after stratification. (B) Early seedling growth: germinating seeds of the wild-type Col, *soar1-2* and *soar1-3* mutants, and four *SOAR1* overexpression lines (OE1, OE3, OE6, and OE12) were transferred, 48h after stratification, from ABA-free MS medium to the MS medium supplemented with 0 (left), 200 (middle), or 500 μM (±)ABA, and the growth was investigated 2 weeks after the transfer. (C) Immunoblot analysis of the SOAR1–GFP fusion protein (95kDa) and SOAR1 protein (68kDa) in the 10-day-old seedlings of the wild-type Col and transgenic lines OE2, OE4, OE5, and OE7–OE12, of which the ABA-related phenotypes are presented in B (OE12) and Supplementary Fig. S5 at *JXB* online. Actin was used as a loading control.

*SOAR1*-overexpressing (OE) lines, in which *SOAR1* was fused with *GFP*, were also generated. Immunoblot analysis detected both the natural SOAR1 and the SOAR1–GFP fusion protein in these OE lines (Fig. 1B; Supplementary Fig. S5B at *JXB* online). The OE lines showed strong ABA-insensitive phenotypes in ABA-induced seed germination inhibition and post-germination growth arrest (Figs 1C–E, 2A, B; Supplementary Figs S5A, S6A–D). The intensity of ABA-insensitive phenotypes of the OE lines was much stronger than that of the *abi1-1* dominant mutant, *abi4* and *abi5* loss-of-function mutants, and a strong *ABI2*-overexpressing line ABI2-OE (Figs 1C–E and 2A, B; Supplementary Figs S5A, S6A–D), all of which are well-characterized strong ABA-insensitive mutants (Gosti *et al.*, 1999; Leung *et al.*, 1997; Finkelstein *et al.*, 1998; Finkelstein and Lynch, 2000; Sun *et al.*, 2011). It is noteworthy that the seeds of the *SOAR1* OE lines germinated and their post-germination seedlings continued to grow in the medium containing >200 μM (±)ABA, while the wild-type Col seeds generally do not germinate if the medium contains >3 μM (±)ABA, and the strong ABA-insensitive ABI2-OE line did not grow in the 100 μM (±)ABA-containing medium (Fig. 2A; Supplementary Figs. S5A, S6A–D available at *JXB* online). The 48-hour-old germinating seeds of the *SOAR1* OE lines even grew in the medium containing >500 μM (±)ABA (Fig. 2B). These data showed that SOAR1 up-regulation almost completely abolished ABA responses of seeds and young seedlings, indicating the critical role of SOAR1 in ABA signalling.

The *GFP* transgenic lines showed wild-type phenotypes (Supplementary Fig. S5C at *JXB* online), revealing that the ABA-related phenotypes of the transgenic *SOAR1-GFP* fusion lines were specific. The complementation lines of the *soar1-2* and *soar1-3* mutants rescued the ABA-hypersensitive phenotypes of these mutants (Supplementary Fig. S7), demonstrating that the mutation in the *SOAR1* gene is responsible for the altered ABA responses of the mutants. Additionally, it was shown that ABA concentrations were not significantly changed in the *soar1-2* mutant and the *SOAR1*-overexpressiing line OE1 compared with the wild-type plants (Supplementary Fig. S8), revealing that ABA metabolism is not significantly affected by changes in *SOAR1* expression.

### SOAR1 *overexpression suppresses ABA hypersensitive phenotypes of the* ABAR *overexpression lines*

**Fig. 3.**
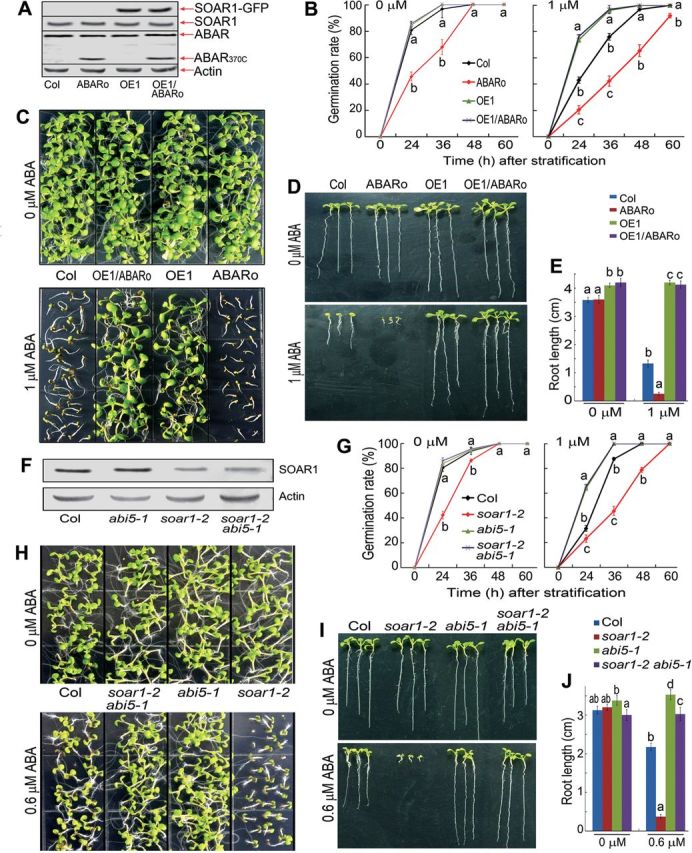
SOAR1 acts genetically downstream of ABAR and upstream of ABI5 in ABA signalling. (A) Immunoblot analysis of the SOAR1–GFP fusion, SOAR1, ABAR, and truncated ABAR (ABAR_370C_) proteins in 10-day-old seedlings of the wild-type Col, *ABAR* overexpressor (ABARo), *SOAR1* overexpressor (OE1), and ABARo/OE1 double overexpression line. Actin was used as a loading control. (B) Seed germination rates of the different genotypes described in (A), which were recorded on ABA-free (0 μM) and ABA-containing (1 μM) MS medium from 24h to 60h after stratification. (C–E) Early seedling growth of the different genotypes described in (A). Seeds were directly planted in MS medium supplemented with 0 or 1 μM (±)ABA; the growth was investigated (C and D) and root length (E) was measured 10 d after stratification. (F) Immunoblot analysis of the SOAR1 protein in 10-day-old seedlings of the wild-type Col, *abi5-1*, *soar1-2* mutants, and the *soar1-2 abi5-1* double mutant. Actin was used as a loading control. (G) Seed germination rates of the different genotypes described in (F), which were recorded on ABA-free (0 μM) and ABA-containing (1 μM) MS medium from 24h to 60h after stratification. (H–J) Early seedling growth of the different genotypes described in (F). Seeds were directly planted in MS medium supplemented with 0 or 0.6 μM (±)ABA; the growth was investigated (H and I) and root length (J) was measured 10 d after stratification. Each value in (B), (E), (G), and (J) is the mean ±SE of five biological determinations, and different letters indicate significant differences at *P*<0.05 (Duncan’s multiple range test) when comparing the germination rates among different genotypes at the same time point after stratification (B, G) or comparing the root lengths among different genotypes in the ABA-free and ABA-containing medium (E, J).

The *SOAR1* and *ABAR* double overexpression lines were generated by using an *ABAR* overexpression line that expressed a truncated ABAR and showed ABA-hypersensitive phenotypes as described previously (Wu *et al.*, 2009). Immunoblot analysis detected the truncated ABAR and SOAR1–GFP fusion proteins in the *SOAR1* and *ABAR* double overexpression lines (Fig. 3A). These double overexpression lines showed strong ABA-insensitive phenotypes in ABA-induced seed germination inhibition and post-germination growth arrest, which were similar to the *SOAR1* overexpression lines (Fig. 3B–E). These findings, together with the discovery of the *soar1-1D* dominant mutant (Fig. 1A, B), reveal that SOAR1 functions downstream of ABAR in the ABA signalling pathway.

### *The* SOAR1 *gene is expressed in the whole plant but preferentially in seeds, and the SOAR1 protein is localized to both the cytosol and nucleus*

The gene expression data in the public websites at http://bar.utoronto.ca and http://www.genevestigator.com showed that the *SOAR1* gene is expressed in different organs/tissues, with the highest level in seeds, and this expression profile was confirmed with the *SOAR1* promoter–*GUS* transgenic lines (Supplementary Fig. S9 at *JXB* online).

**Fig. 4.**
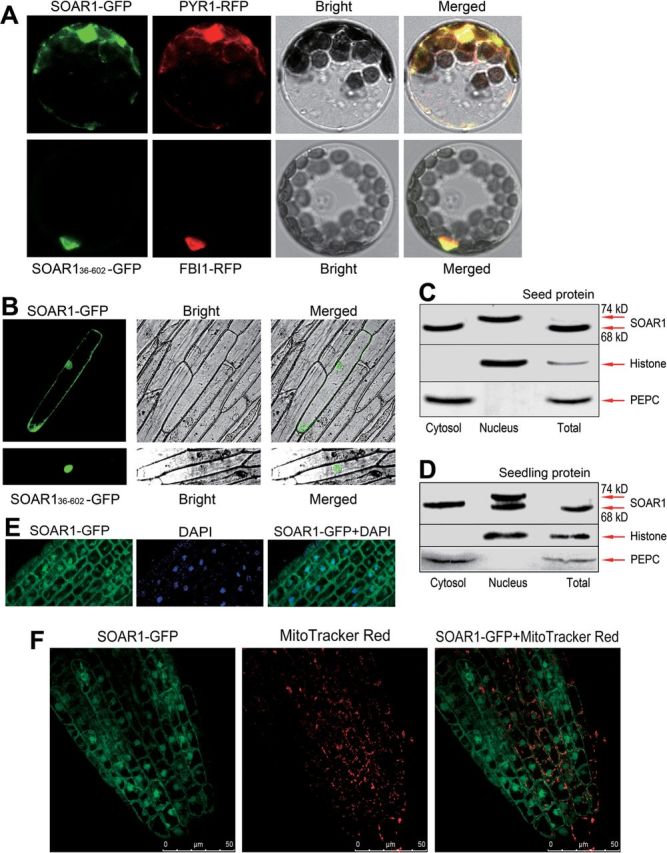
SOAR1 is localized in both the cytosol and nucleus. (A) Transient expression in *Arabidopsis* protoplasts. Top panels: SOAR1 is co-localized with a nuclear–cytosol-localized protein PYR1 (At4g17870; Park *et al.*, 2009). SOAR1 was tagged with green fluorescent protein (SOAR1–GFP), and PYR1 protein was tagged with mCherry (PYR1–RFP). Bottom panels: the N-terminal truncated SOAR1 (SOAR1_36–602_, with 35 amino acid residues deleted at the N-terminus of the SOAR1 protein; see Supplementary Fig. S1A at *JXB* online) is co-localized with a nuclear-localized FBI1 bHLH transcription factor (At1g02340; Fairchild *et al.*, 2000; Jang *et al.*, 2005). SOAR1_36–602_ was tagged with GFP (SOAR1_36–602_–GFP), and FBI1 protein was tagged with mCherry (FBI1–RFP). Bright, bright-field; Merged, merged image. The experiments were repeated five times with the same results. (B) Transient expression of the above-mentioned constructs in onion epidermis cells. (C) and (D) Immunoblot analysis of the SOAR1 protein in the total protein (Total), cytosolic (Cytosol), and nuclear (Nucleus) fractions from the seeds sampled 24h after stratification (C) and 10-day-old seedlings (D). Histone H3 (nuclear marker) and PEPC (phosphoenolpyruvate carboxylase; cytosolic marker) were tested in the cytosolic and nuclear fractions to estimate the purity of the fractions. (E) Transgenic expression of the SOAR1–GFP fusion protein in *Arabidopsis* whole plants. A portion of the SOAR1–GFP fusion protein (left) and a nuclear marker DAPI (middle) are co-localized to the nucleus (right, SOAR1–GFP+DAPI) in the root of the transgenic line OE1. The experiments were repeated five times with the same results. (F) Transgenic expression of the SOAR1–GFP fusion protein in *Arabidopsis* whole plants. Investigations were performed in the root of the OE1 line. SOAR1–GFP, the distribution pattern of SOAR1–GFP fusion protein (left); Mito Tracker Red, the profile of mitochondria stained by Mito Tracker Red (middle); SOAR1–GFP+Mito Tracker Red, merged image of SOAR1–GFP and Mito Tracker Red (right). The images show that SOAR1–GFP localization is distinct from the Mito Tracker Red-stained mitochondrial profile. The experiments were repeated five times with the same results.

As regards the subcellular localization of SOAR1, a bioinformatics search allowed the prediction that SOAR1 may localize to the mitochondrion, chloroplast, or nucleus (Supplementary Fig. S10 at *JXB* online). The transient expression assays in *Arabidopsis* protoplasts showed that SOAR1 co-localized with the cytosol–nucleus dual-localized PYR1 (Fig. 4A), which is a member of the PYR/PYL/RCAR receptors for ABA (Ma *et al.*, 2009; Park *et al.*, 2009; Santiago *et al.*, 2009), while the SOAR1 fluorescence was not seen in the chloroplasts (Fig. 4A). However, the cytosolic SOAR1 disappeared, and the SOAR1 fluorescence was seen only in the nucleus and co-localized with a nuclear marker, bHLH (basic helix–loop–helix) transcription factor FBI1 (At1g02340; Fairchild *et al.*, 2000; Jang *et al.*, 2005) when an N-terminal 35 amino acid fragment was deleted (Fig. 4A), suggesting that the N-terminal 35 amino acid fragment is required for the cytosolic localization of the SOAR1 protein. The data from the transient expression assays in onion epidermis cells are consistent with those from the transgenic *Arabidopsis* protoplasts (Fig. 4B).

The localization of SOAR1 was further verified by immunoblot assays with the purified cytosolic and nuclear fractions from *Arabidopsis*, in which the cytosolic marker could not be detected in the nuclear fraction and the nuclear marker histone H3 could not be detected in the cytosolic fraction (Fig. 4C, D), showing the purity of the cytosolic and nuclear fraction. Using the antibody specific for SOAR1 (Supplementary Fig. S3 at *JXB* online), we immunodetected the SOAR1 protein in both the purified cytosolic and nuclear fractions (Fig. 4C, D), which consistently confirmed that SOAR1 is a cytosol and nucleus dual-localized protein.

It is noteworthy that, in the proteins sampled from the germinating seeds 24h after stratification, a form of SOAR1 protein was detected in the nuclear fraction with a higher molecular mass (74kDa) than the normal protein in the cytosolic fraction (68kDa) (Fig. 4C); and in the proteins sampled from the 2-week-old seedlings, both forms of SOAR1 protein were detected in the nuclear fraction with molecular masses of 68kDa and 74kDa, respectively (Fig. 4D). However, only a very weak signal of the 74kDa protein was detected that often could be scarcely seen in the total proteins (Fig. 4C, D) probably because of too low a concentration of the SOAR1 in the total protein extracts. These data indicate that the SOAR1 protein is subjected to a post-translational modification before or after it enters the nucleus, which may be associated with its function in the nucleus. This aspect remains an open question and needs further studies in the future.

Given that SOAR1 protein is present in the cytosolic space surrounding the mitochondria, it was further tested whether SOAR1 resides in the mitochondrion by using the above-mentioned *SOAR1-GFP* transgenic line OE1 (Fig. 1A). The nuclear localization of the SOAR1–GFP fusion protein was first verified in the root of the OE1 plants, as visualized by co-localization of the GFP fluorescence with the DAPI-stained nuclei (Fig. 4E). It was further shown that the localization pattern of the SOAR1 protein visualized by GFP fluorescence is distinct from the distribution profile of the mitochondria labelled by a mitochondrial marker (MitoTracker Red) (Fig. 4F). Taken together, these data demonstrate that SOAR1 localizes to the cytosol and nucleus, but not to chloroplasts or mitochondria.

### *Changes in* SOAR1 *expression alter expression of a subset of ABA-responsive genes*

**Fig. 5.**
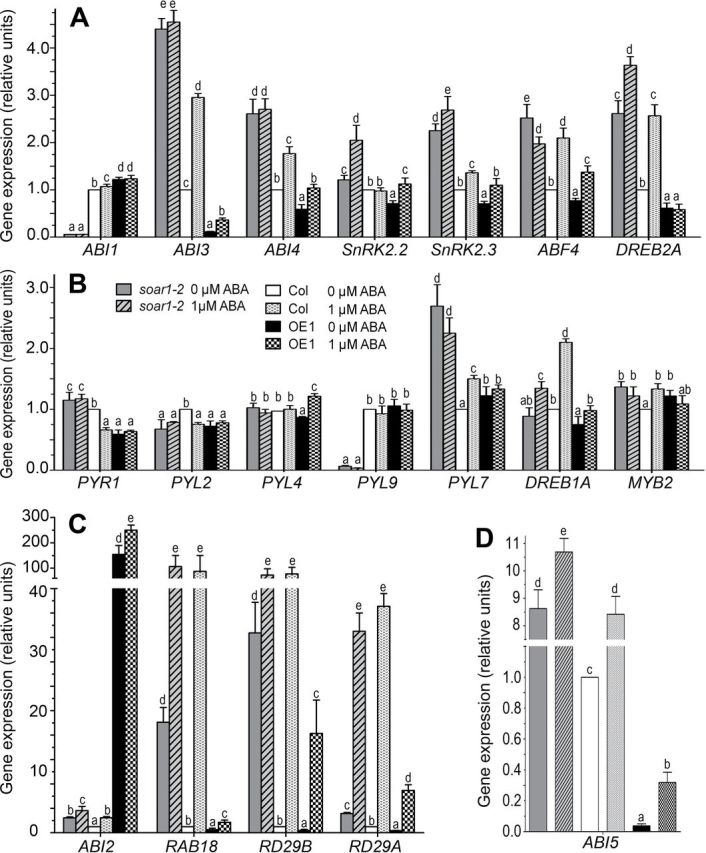
Down- or up-regulation of *SOAR1* alters expression of a subset of genes involved in ABA signalling. The RNA sample was extracted from the germinating seeds 24h after a 3 d stratification, and the gene expression levels were analysed by quantitative real-time PCR (A–D). The gene expression levels were relative units normalized relative to the value from the sample of the wild-type Col plants (as 1). Each column is the same in all panels, and a key is provided in B. Each value is the mean ±SE of three biological determinations, and different letters indicate significant differences at *P*<0.05 (Duncan’s multiple range test) when comparing the expression levels for the same gene among different genotypes treated with the ABA-free (0 μM) or 1 μM ABA-containing solution.

The expression levels of a subset of the ABA-responsive genes were tested in the *soar1-2* mutant and the *SOAR1* overexpressor OE1. These genes include *ABF4/AREB2* (Choi *et al.*, 2000; Uno *et al.*, 2000), *ABI1* (Leung *et al.*, 1994; Meyer *et al.*, 1994; Gosti *et al.*, 1999), *ABI2* (Leung *et al.*, 1997), *ABI3* (Giraudat *et al.*, 1992), *ABI4* (Finkelstein *et al.*, 1998), *ABI5* (Finkelstein and Lynch, 2000), *DREB1A*, *DREB2A* (Liu *et al.*, 1998), *MYB2* (Abe *et al.*, 2003), *PYR1/RCAR11*, *PYL2/RCAR14*, *PYL4/RCAR10*, *PYL7/RCAR2*, *PYL9/RCAR1* (Ma *et al.*, 2009; Park *et al.*, 2009), *RD29A*, *RD29B* (Yamaguchi-Shinozaki and Shinozaki, 1994), *RAB18* (Lang and Palva, 1992), *SnRK2.2*, and *SnRK2.3* (Fujii *et al.*, 2007; Fujii and Zhu, 2009). The expression of the positive ABA signalling regulator-encoding genes (or positively ABA-responsive genes) *ABI3*, *ABI4*, *ABI5*, *ABF4*, *DREB2A*, *PYR1*, *RAB18*, *RD29A*, *RD29B*, *SnRK2.2*, and *SnRK2.3* was significantly up-regulated in the *soar1-2* mutant, while it was repressed in the OE1 line (Fig. 5A–D). However, the expression levels of the other positive ABA signalling regulator-encoding genes *PYL2*, *PYL4*, *DREB1A*, and *MYB2* was not changed much in the *soar1-2* mutant and the OE1 line compared with the wild-type plants, though some significant differences were still detected (Fig. 5B). The expression of another two genes encoding ABA receptors was altered differently: *PYL9* was remarkably repressed, while *PYL7* was significantly up-regulated in the *soar1-2* mutant (Fig. 5B). It is noteworthy that the *ABI3* and *ABI5* genes, encoding two critical, positive regulators of ABA-responsive seed germination and post-germination growth, were substantially suppressed to a null level in the OE1 line while they were considerably up-regulated in the *soar1-2* mutant (Fig. 5A, D), suggesting that these genes are potential, main targets of the SOAR1 protein. The expression of the negative ABA signalling regulator-encoding gene *ABI1* was significantly repressed in the *soar1-2* mutant, while *ABI2* was remarkably up-regulated in the OE1 line (Fig. 5A, C).

The exogenous ABA treatment enhanced the expression levels of the ABA-responsive genes including *ABI1*, *ABI2*, *ABI3*, *ABI4*, *ABF4*, *DREB1A*, *DREB2A*, *MYB2*, *PYL7*, *RAB18*, *RD29A*, *RD29B*, and *SnRK2.3* in the wild-type Col plants, whereas such ABA responsiveness of gene expression was significantly altered in the *soar1-2* mutant and OE1 line (Fig. 5A–C). It is noteworthy that, with the ABA treatment, the expression levels of *ABI3* and *ABI5* still remained lower in the OE1 line (Fig. 5A, D), and the levels of most of the positive ABA signalling regulator-encoding genes such as *ABI3*, *ABI4*, *ABI5*, *ABF4*, *DREB2A*, *PYL7*, *SnRK2.2*, and *SnRK2.3* remained higher in the *soar1-2* mutant than in wild-type plants (Fig. 5A–D). The low expression of *ABI1* was not significantly changed by ABA treatment in the *soar1-2* mutant, and an even higher level of *ABI2* was observed in the ABA-treated OE1 line (Fig. 5A, C).

Overall, these gene expression data are globally consistent with ABA-insensitive phenotypes of the *SOAR1* overexpression lines and ABA-hypersensitive phenotypes of the *soar1-2* and *soar1-3* mutants. In particular, the remarkably high level of *ABI2*, together with low levels of *ABI3* and *ABI5* in the OE1 line, may be linked directly to the strong ABA-insensitive phenotypes of the *SOAR1* overexpression lines, and the significantly high levels of *ABI3*, *ABI5*, *ABF4*, *PYR7*, and *SnRK2.3* and the low level of *ABI1* may be associated directly with the ABA-hypersensitive phenotypes of the *soar1-2* and *soar1-3* mutants (Figs 1, 2; Supplementary Figs S4–S7 at *JXB* online). Additionally, the expression profile of the ABA-responsive genes in the *soar1-2* mutant and OE1 line suggests the presence of a complicated feed-forward and feed-back mechanism that may balance positive and negative regulation of gene expression to optimize ABA signalling.

### *Loss of function of* ABI5 *suppresses ABA-hypersensitive phenotypes of the* soar1-2 *T-DNA insertion mutant*

Given that the ABI5 transcription factor is a key player regulating seed germination and post-germination growth in response to ABA (Finkelstein and Lynch, 2000; Lopez-Molina *et al.*, 2001), and the *ABI5* gene expression is significantly up-regulated in the *soar1-2* mutant and nearly knocked out in the OE1 line (Fig. 5C), it was tested whether ABI5 functions downstream of SOAR1 as a potential target of this PPR protein. The *abi5-1 soar1-2* double mutant was generated, in which *abi5-1* is a knockout allele of the *ABI5* gene and *soar1-2* a knockdown allele of the *SOAR1* gene. The *abi5-1 soar1-2* double mutant showed ABA-insensitive phenotypes in ABA-induced seed germination inhibition and post-germination growth arrest, which are similar to the *abi5-1* mutant (Fig. 3F–J). These findings suggest that SOAR1 may function upstream of ABI5 in the ABA signalling pathway.

## Discussion

### SOAR1 is a critical, negative, regulator acting downstream of ABAR and probably upstream of ABI5 in ABA signalling

It was shown that down-regulation of *SOAR1* strongly increases, and up-regulation of *SOAR1* almost completely impairs, ABA sensitivity in seed germination and post-germination growth. The intensity of the ABA overly-sensitive phenotypes of the two mutants was similar to, or stronger than, that of the well-characterized *abi1 abi2* double-knockout mutant (Fig. 1; Supplementary Fig. S4 at *JXB* online). It could be expected that a complete loss of SOAR1 function would lead to even stronger ABA-hypersensitive phenotypes or dormant seeds; however, the null allele of the *SOAR1* gene is likely to be lethal. The intensity of ABA-insensitive phenotypes of the *SOAR1*-overexpressing lines was much stronger than that of the *abi1-1* dominant mutant, *abi4* and *abi5* loss-of-function mutants, and a strong *ABI2*-overexpressing line ABI2-OE (Figs 1, 2; Supplementary Figs S5, S6). In particular, it is surprising to note that the seeds of the *SOAR1* overexpressors germinated and their post-germination seedlings continued to grow in the medium containing >200 μM (±)ABA (Fig. 2; Supplementary Figs S5, S6), and the 48-hour-old germinating seeds of the *SOAR1* overexpressors even grew in the medium containing >500 μM (±)ABA (Fig. 2). Previous studies reported that the seeds of the *srk2dei* mutant, a triple-knockout mutant of three SnRK2 members SnRK2.2, SnRK2.3, and SnRK2.6, germinated and continued to grow in the presence of 50 μM or 100 μM exogenous ABA, which is believed to impair the ABA response completely (Fujii and Zhu, 2009; Nakashima *et al.*, 2009; Umezawa *et al.*, 2009). In this regard, the intensity of ABA insensitivity of the *SOAR1* overexpression lines in this study is comparable with that of the triple loss-of-function mutant of the SnRK2 members (Fig. 2; Supplementary Figs S5, S6). However, the seeds of the *SOAR1* overexpression lines are not viviparous despite their strong ABA insensitivity, suggesting that there may be a sophisticated mechanism to balance and optimize the function of SOAR1 under basal growth conditions.

All the findings reveal that SOAR1 is a critical, negative, regulator of ABA signalling, which regulates key processes of cell signalling in response to ABA. The ABA hypersensitivity of the *ABAR* overexpressors was suppressed by up-regulation of *SOAR1* (Figs 1, 5), showing that SOAR1 functions in ABA signalling downstream of ABAR. Loss of function of *ABI5* suppressed ABA-hypersensitive phenotypes of a *SOAR1* knockdown mutant (*soar1-2*), suggesting that SOAR1 may function upstream of ABI5. These findings suggest a possible ABAR–SOAR1–ABI5-linked signalling cascade in the ABA signalling pathway, though it still remains unknown whether a direct interconnection exists between ABAR and SOAR1 or between SOAR1 and ABI5.

### How does SOAR1 work in ABA signalling?

Currently, it remains largely unknown whether and how the PPR proteins regulate nuclear gene expression. In the two identified *Arabidopsis* nucleus-localized PPR proteins, GRP23 interacts physically with RNA polymerase II, suggesting its potential function as a transcription regulator (Ding *et al.*, 2006); PNM1, dual localized to both the nucleus and mitochondrion, physically interacts with the nucleosome assembly protein NAP1 and the transcription factor TCP8, suggesting its potential roles in the coordination of mitochondrial and nuclear gene expression (Hammani *et al.*, 2011). However, whether and how these nuclear PPR proteins regulate nuclear gene expression, and what their downstream, nuclear target genes are, have not been reported (Ding *et al.*, 2006; Hammani *et al.*, 2011).

In the present experiments, it was observed that down-regulation of the *SOAR1* gene enhanced, but up-regulation of the *SOAR1* gene repressed, the expression levels of the ABA-responsive genes *ABI3*, *ABI4*, *ABI5*, *ABF4*, *DREB2A*, *PYR1*, *RAB18*, *RD29A*, *RD29B*, *SnRK2.2*, and *SnRK2.3*. It is particularly noteworthy that the expression levels of the *ABI3*, *ABI5*, *RAB18*, and *RD29B* genes were markedly increased, while the level of *ABI1* was almost completely suppressed by down-regulation of the *SOAR1* gene, and the *ABI3* and *ABI5* genes were almost completely suppressed by overexpression of the *SOAR1* gene (Fig. 5). It is also surprising to observe that the level of *ABI2* expression was increased by >100-fold with up-regulation of the *SOAR1* gene (Fig. 5). The marked increase of the *ABI2* mRNA in the *SOAR1* overexpression lines is likely to be caused by a decrease of some repressive factors of *ABI2* to which is SOAR1 targeted, protecting the *ABI2* mRNA from degradation. An ABA receptor member-encoding gene *PYL9* was repressed, while the gene of another member *PYL7* was up-regulated in the *soar1-2* mutant (Fig. 5), suggesting a SOAR1-related balance mechanism that may function in RNA processing to maintain homeostasis of these family proteins. These gene expression data strongly suggest that these genes are most probably potential, direct or indirect targets of the SOAR1 protein, and explain, at least partly, the strong ABA-related phenotypes of the *soar1* mutants and *SOAR1* overexpressors. The gene expression profile under treatment with exogenous ABA is consistent with this conclusion. Genetic evidence that ABI5 may function downstream of SOAR1 (Fig. 3) strongly supports that *ABI5* mRNA may be a target of the SOAR1 protein, which may function as a SOAR1–ABI5 directly coupled signalling module in the ABA signalling pathway.

It remains largely unclear whether SOAR1 participates in the PYR/PYL/RCAR-mediated ABA signalling pathway, a well-characterized core ABA signalling pathway (Fujii *et al.*, 2009; Ma *et al.*, 2009; Park *et al.*, 2009; Santiago *et al.*, 2009; Cutler *et al.*, 2010). However, the expression data of the ABA-responsive genes in the *soar1-2* mutant and *SOAR1* overexpressor OE1 (Fig. 5) showed that the alteration in *SOAR1* expression significantly changes the expression of a subset of genes, of which the encoded proteins have been identified to be directly involved in the PYR/PYL/RCAR-mediated ABA signalling, including the PYR/PYL/RCAR family members PYR1/RCAR11, PYL7/RCAR2, and PYL9/RCAR1, and some key signalling components ABI1, ABI2, SnRK2.2, SnRK2.3, ABI5, and ABF4 (Fig. 5). If the *ABI5* mRNA is a direct target of SOAR1, ABI5 may be a common target of SOAR1 and SnRK2.2/3/6 in the ABA signalling pathway where the SnRK2 members regulate ABI5 by a phosphorylation process, a post-translation modification (Fujii *et al.*, 2007; Fujii and Zhu, 2009), while SOAR1 may regulate ABI5 by post-trancriptional RNA processing. Thus, they may cooperate to regulate ABA signalling. Additionally, the gene expression data suggest that SOAR1 may regulate RNA processing of other key signalling component-encoding genes including *SnRK2.2* and *SnRK2.3*. All the gene expression data support the idea that SOAR1 may also be involved in the PYR/PYL/RCAR-mediated ABA signalling which may possibly interact with ABAR/CHLH-mediated signalling through SOAR1. However, whether and how PYR/PYL/RCAR function as ABA receptors to regulate SOAR1, and how PYR/PYL/RCAR-mediated signalling interacts with ABAR/CHLH-mediated signalling through SOAR1, need further studies. Exploration of the detailed mechanisms of the cytosol–nuclear dual-localized SOAR1 protein that functions in the nuclear events as a critical component of ABA signalling, such as the nuclear mechanism by which SOAR1 regulates *ABI5* mRNA processing, will be of particular importance in the future to understand the functional mechanism of PPR proteins and the highly complicated ABA signalling network.

## Supplementary data

Supplementary data are available at *JXB* online.

Figure S1. Diagrams of the three T-DNA insertion mutants in the *SOAR1* gene (At5g11310).

Figure S2. SOAR1 is a member of the pentatricopeptide repeat protein family.

Figure S3. Test of the specificity of the anti-SOAR1 serum.

Figure S4. The *soar1-2* and *soar1-3* mutants are hypersensitive to ABA in seed germination and early seedling growth.

Figure S5. The ABA-insensitive phenotypes in early seedling growth of 12 *SOAR1* overexpression lines.

Figure S6. Phenotypes of the *soar1-1D*, *soar1-2*, and *soar1-3* mutants, and *SOAR1* and *ABI* overexpression lines in response to ABA.

Figure S7. Transgenic expression of *SOAR1* rescues the ABA-hypersensitive phenotypes of the *soar1-2* and *soar1-3* mutant.

Figure S8. ABA concentrations in the germinating seeds of different genotypes.

Figure S9. *SOAR1* is expressed in different organs/tissues, with the highest expression level in seeds.

Figure S10. Prediction of the subcellular localization of SOAR1 protein.

Table S1. Primers used in this study.

Supplementary Data
