# Supplementary material for: Arabidopsis pentatricopeptide repeat protein SOAR1 plays a critical role in abscisic acid signalling
Source: J Exp Bot. 2014 Jul 8;65(18):5317–30. doi: 10.1093/jxb/eru293 (PMC4157714; doi:10.1093/jxb/eru293)
Supplement: Supplementary Data [file supp_65_18_5317__index.html]

 Arabidopsis pentatricopeptide repeat protein SOAR1 plays a critical role in abscisic acid signalling — Arabidopsis pentatricopeptide repeat protein SOAR1 plays a critical role in abscisic acid signalling — Supplementary Data 

# *Arabidopsis* pentatricopeptide repeat protein SOAR1 plays a critical role in abscisic acid signalling

## Supplementary Data

Data files

**Files in this Data Supplement:**

- Supplementary Data - Supplementary Data
