# Supplementary material for: Arabidopsis pentatricopeptide repeat protein SOAR1 plays a critical role in abscisic acid signalling
Source: J Exp Bot. 2014 Jul 8;65(18):5317–30. doi: 10.1093/jxb/eru293 (PMC4157714; doi:10.1093/jxb/eru293)
Supplement: Supplementary Data [file supp_eru293_jexbot127035_file001.pdf]

Supplementary Data

Mei & Jiang *et al*., *Arabidopsis* pentatricopeptide repeat protein SOAR1 plays a critical role in abscisic acid signaling

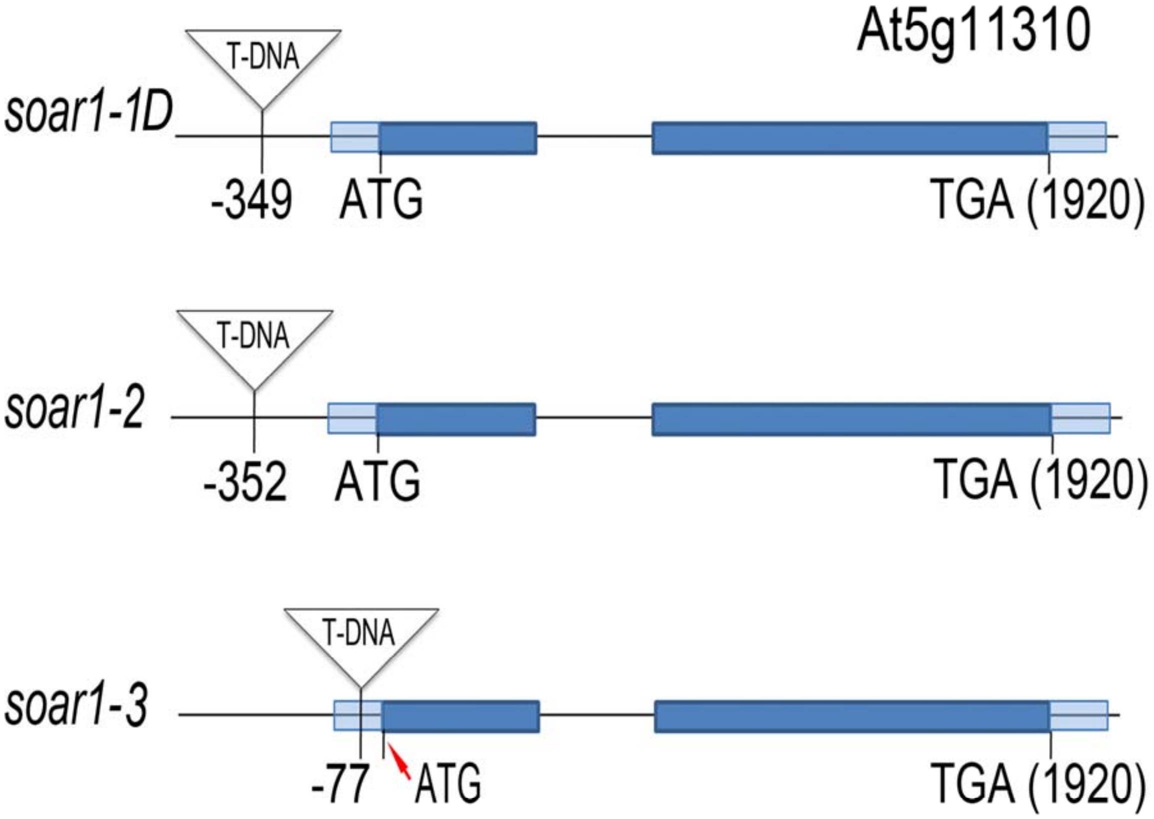

Supplementary Figure S1. Diagrams of the Three T-DNA Insertion Mutants in the *SOAR1* Gene (At5g11310). The *soar1-1D*, *soar1-2* and *soar1-3* mutants are mutants of T-DNA insertion in the promoter region of the At5g11310 locus at 349, 352, and 77 bp upstream of the start codon (ATG), respectively.

A

MNSLFTAFRRNLLLNPNPHRNFFLHRLLSSSRRSSPLIPVEPLIQRIQSPAVPDSTCTPPQQNTVSKTDLSTISNLL
ENTDVVPGSSLESALDETGIEPSVELVHALFDRLSSSPMLLHSVFKWAEMKPGFTLSPSLFDSVVNSLCKAREFEIA
WSLVFDRVRSDEGSNLVSADTFIVLIRRYARAGMVQQAIRAFEFARSYEPVCKSATELRLLEVLLDALCKEGHVREA
SMYLERIGGTMDSNWVPSVRIFNILLNGWFRSRKLKQAEKLWEEMKAMNVKPTVVTYGTLIEGYCRMRRVQIAMEVL
EEMKMAEMEINFMVFNPIIDGLGEAGRLSEALGMMERFFVCESGPTIVTYNSLVKNFCKAGDLPGASKILKMMMTRG
VDPTTTTYNHFFKYFSKHNKTEEGMNLYFKLIEAGHSPDRLTYHLILKMLCEDGKLSLAMQVNKEMKNRGIDPDLLT
TTMLIHLLCRLEMLEEAFEEFDNAVRRGIIPQYITFKMIDNGLRSKGMSDMAKRLSSLMSSLPHSKKLPNTYREAVD
APPDKDRRKSILHRAEAMSDVLKGCRNPRKLVKMRGSHKKAVGEDINLIDDINERNGDAGDFE

B

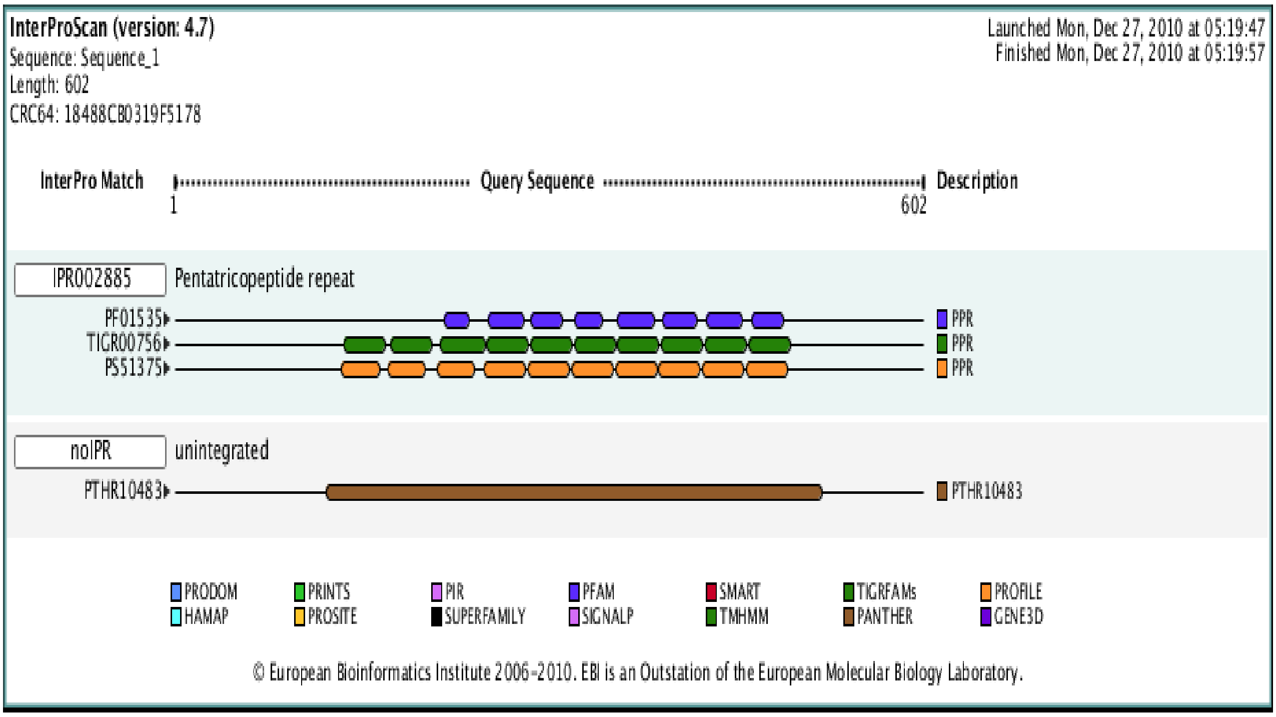

Supplementary Figure S2. SOAR1 Is a Member of the Pentatricopeptide Repeat Protein Family. **(A)** The amino acid sequence and PPR domains of SOAR1: the marked sequences are the PPR domains according to the TIGR00756 (band in green) as mentioned below in (B). The deleted N-terminal amino acid residues in the subcellular localization assay (see Figure 5) are marked in red. **(B)** Prediction of PPR domains of SOAR1 by a website of European Information Institute (http://www.ebi.ac.uk/Tools/InterProScan/index.html).

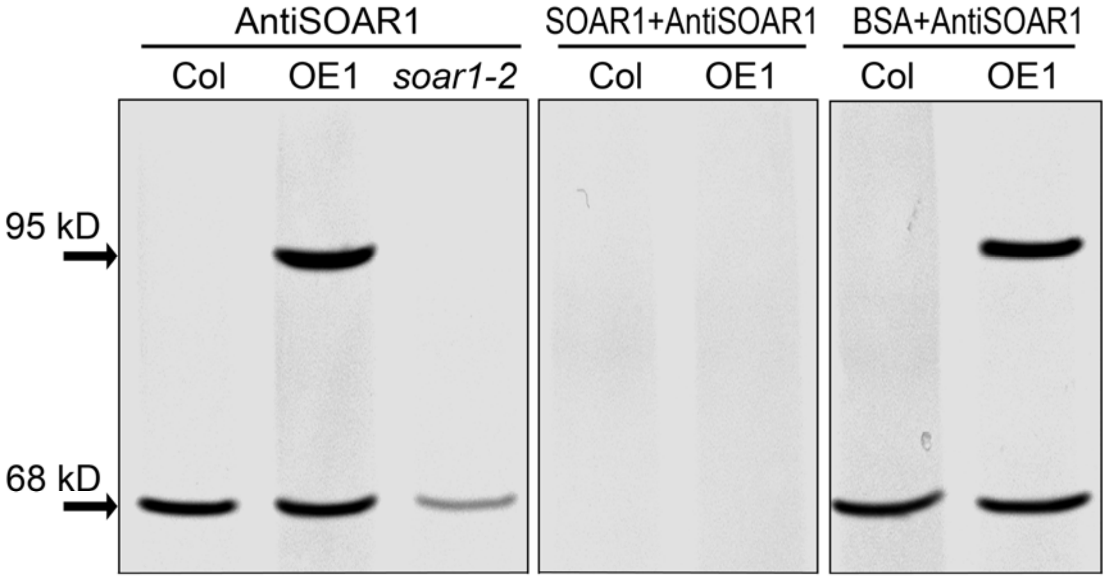

Supplementary Figure S3. Test of the Specificity of the Anti-SOAR1 Serum. The immunoblotting assays were conducted in *Arabidopsis* total protein on a 12% SDS-PAGE gel with equal amount of protein loading sample**. Left panel:** The native SOAR1 protein (68 kD) was recognized by the anti-SOAR1 serum (gels indicated by AntiSOAR1) in the wild-type Col plants, *SOAR1*-overexpression line OE1 and *soar1-2* knockdown mutant with a weaker immuno-signal in the *soar1-2* mutant. The SOAR1-GFP fusion protein was detected by the anti-SOAR1 serum only in *SOAR1*-overexpression line OE1. **Middle panel:** A blocking process of the anti-SOAR1 serum by the *E. coli*-expressed, truncated SOAR1 protein (the anti-SOAR serum was incubated with the truncated SOAR antigen protein at 0℃ for 30 min) eliminated the antiserum reaction with SOAR1 (gels indicated by SOAR1+AntiSOAR1) in the Col plants and OE1 line. **Right panel:** As a control, BSA protein, instead of the truncated SOAR1 antigen protein, was used to incubate the anti-SOAR serum, which did not affect the antiserum reaction with SOAR1 (gels indicated by BSA+AntiSOAR1) in the Col plants and OE1 line. Arrows with “95 kD” and “68 kD” indicate molecular mass (kilodalton) of the SOAR1-GFP fusion and native SOAR1 protein, respectively.

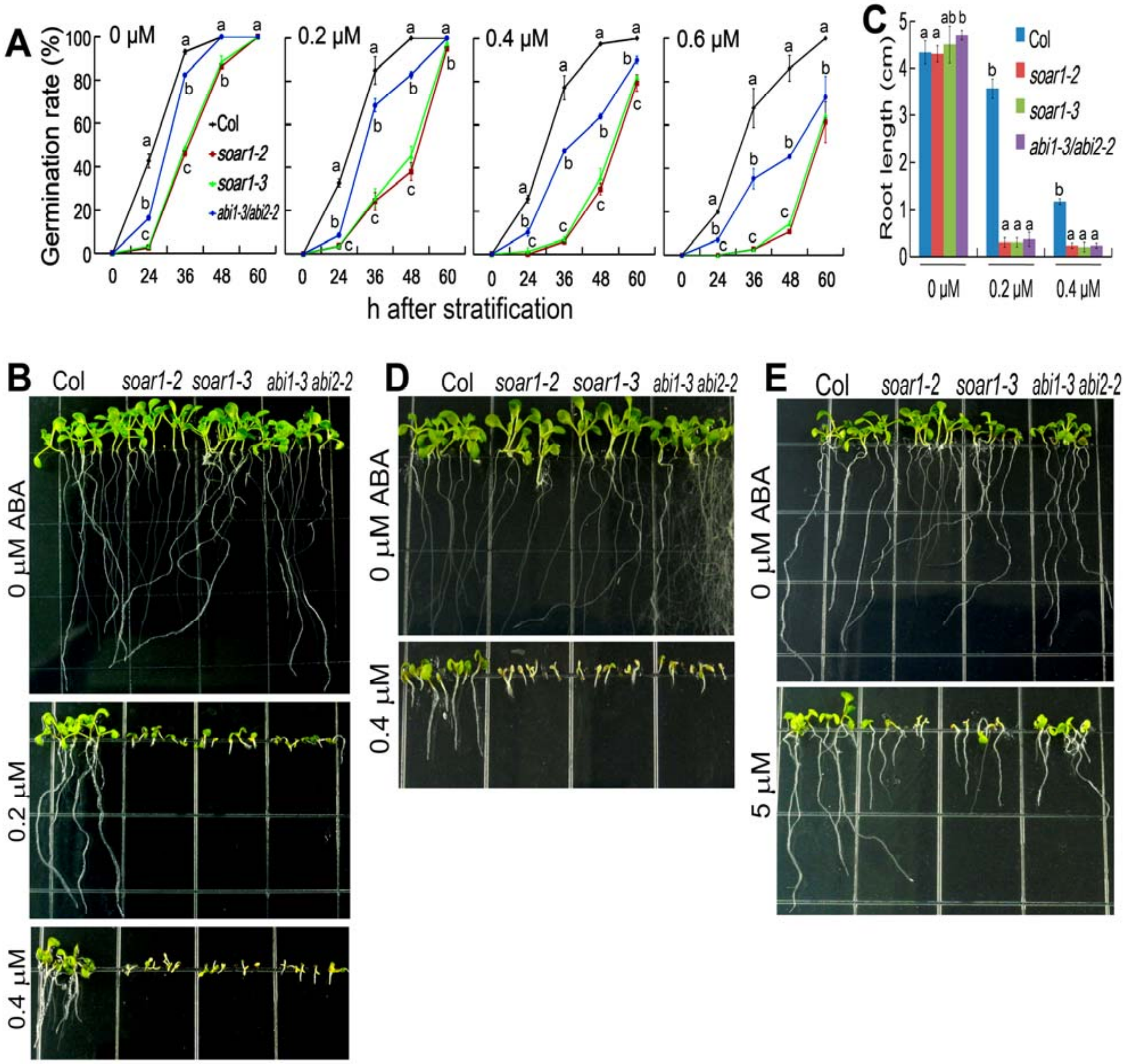

Supplementary Figure S4. The *soar1-2* and *soar1-3* Mutants Are Hypersensitive to ABA in Seed Germination and Early Seedling Growth. **(A)** Seed germination: germination rates of the wild-type Col, *soar1-1*, *soar1-2* mutants and *abi1-3 abi2-2* double-knockout mutant were recorded on ABA-free (0 μM) and ABA-containing (0.2, 0.4 or 0.6 μM) medium from 24 to 60 h after stratification. Each value is the mean ± SE of five biological determinations, and different letters indicate significant differences at $P < 0.05$ (Duncan's multiple-range test) when comparing the germination rates among different genotypes at the same time point after stratification. **(B)** Early seedling growth: seeds from the wild-type Col, *soar1-1*, *soar1-2* mutants and *abi1-3 abi2-2* double-knockout mutant, were directly planted in the MS medium supplemented with 0 (top), 0.2 (middle) or 0.4 μM (±)ABA (bottom), and the growth was investigated 10 d after stratification. **(C)** Statistical values of root growth of different genotypes described in (B). Each value is the mean ± SE of five biological determinations, and different letters

indicate significant differences at $P < 0.05$ (Duncan's multiple-range test) when comparing the root lengths among different genotypes in the ABA-free and ABA-containing medium. **(D)** Early seedling growth: germinating seeds of the wild-type Col, *soar1-1*, *soar1-2* mutants and *abi1-3 abi2-2* double-knockout mutant were transferred, 48 h after stratification, from ABA-free medium to the medium supplemented with 0 (top) or 0.4 μM (±)ABA (bottom), and the growth was investigated 10 d after the transfer. **(E)** Early seedling growth: germinating seeds of the wild-type Col, *soar1-1*, *soar1-2* mutants and *abi1-3 abi2-2* double-knockout mutant were transferred, 4 d after stratification, from ABA-free medium to the medium supplemented with 0 (top) or 5 μM (±)ABA (bottom), and the growth was investigated 10 d after the transfer.

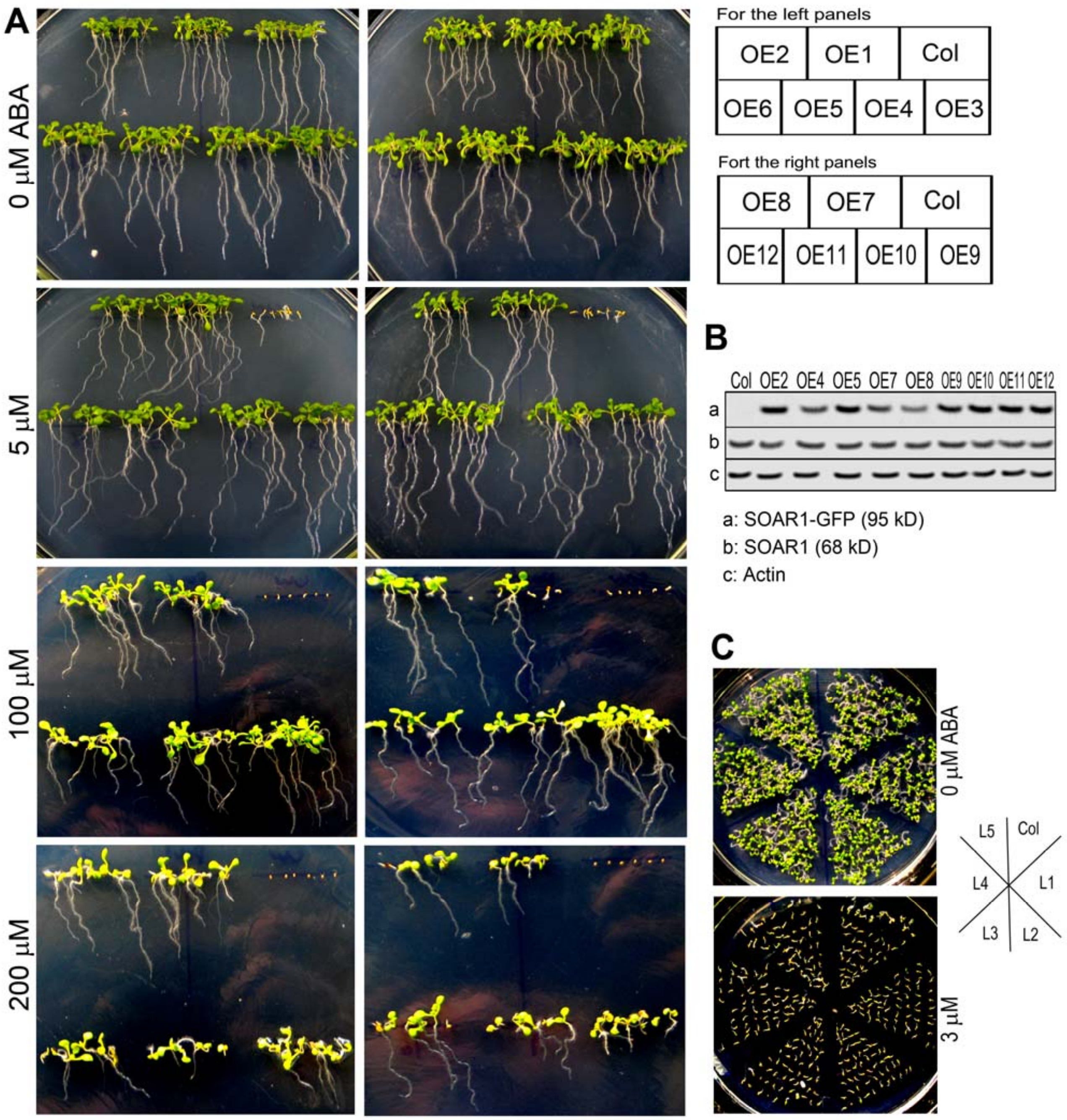

Supplementary Figure S5. The ABA-insensitive Phenotypes in Early Seedling Growth of Twelve *SOAR1*-overexpression Lines. **(A)** Seeds from the wild-type Col and twelve *SOAR*-overexpression lines OE1-OE12, were directly planted in the MS medium supplemented with 0 (top), 5, 100 or 200 μM (±)ABA, and the growth was investigated 10 days after stratification. **(B)** Immunoblot analysis of the SOAR1-GFP fusion and SOAR1 proteins in the ten-day-old seedlings of the wild-type Col and transgenic lines OE2, OE4, OE5 and OE7-OE12. Actin was used as a loading control. These immunoblot data are also shown in Fig. 2C. **(C)** The *GFP*-transgenic lines showed wild-type ABA response. Seeds from the wild-type Col and five *GFP*-transgenic lines L1-L5, were directly planted in the MS medium supplemented with 0 (top) or 3 μM (±)ABA, and the growth was investigated 10 d after stratification.

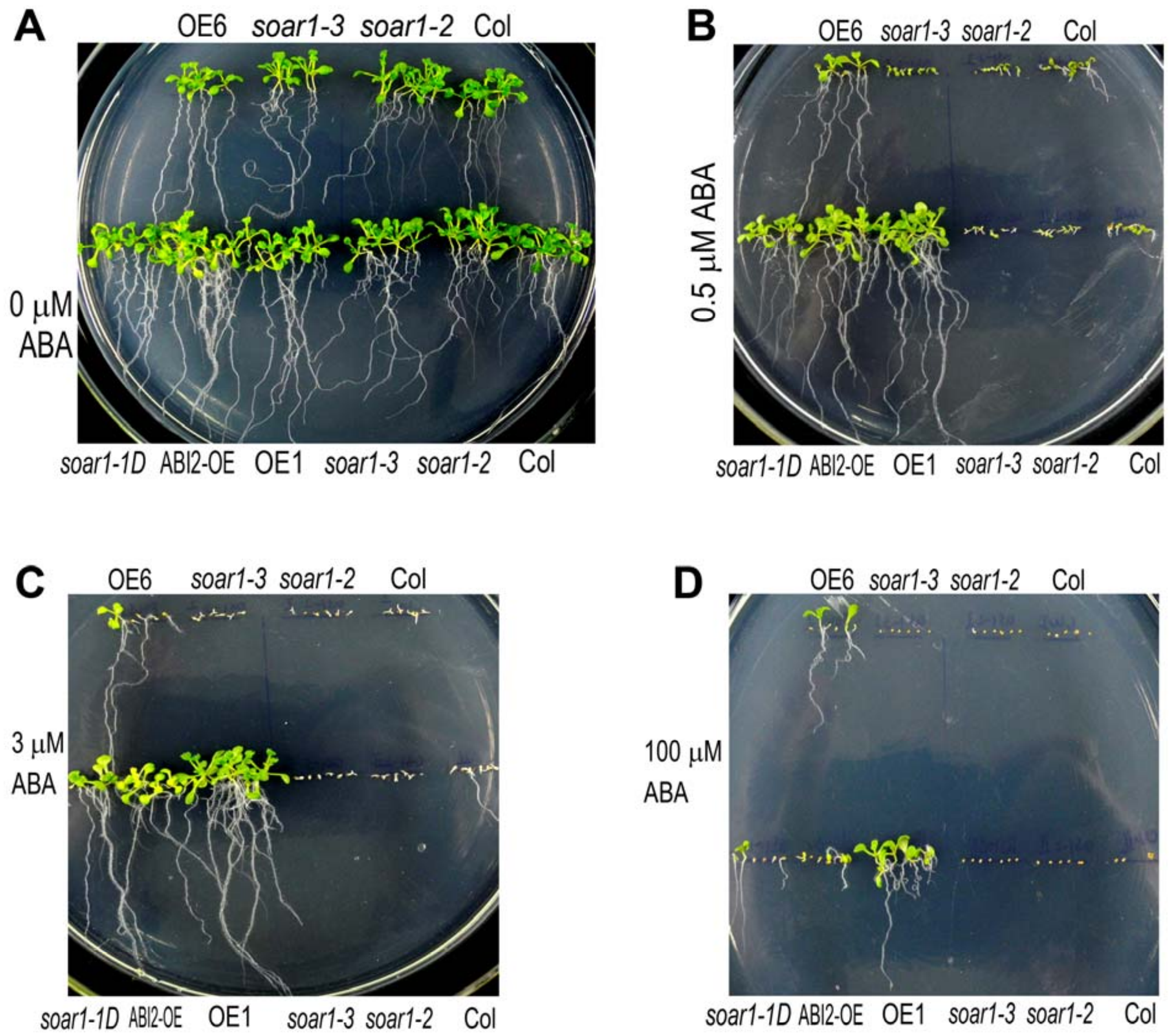

Supplementary Figure S6. Phenotypes of the *soar1-1D*, *soar1-2*, *soar1-3* Mutants and *SOAR1*- and *ABI2*-overexpression Lines in Response to ABA. **(A) – (D)** Seed germination and early seedling growth of the different genotypes in the medium containing 0 (**A**), 0.5 (**B**), 3 (**C**) or 100 μM (**D**) (±)ABA. The seeds were planted directly in the ABA-free or ABA-containing medium, and the growth was investigated 7 d after stratification. The *soar1-2* and *soar1-3* mutants are hypersensitive to ABA, but *SOAR1*-overexpression lines OE1 and OE6 are insensitive to ABA in seed germination and early seedling growth. The ABA-insensitive phenotypes of the two mutant alleles are similar to, or stronger than, the *ABI2*-overexpression line ABI2-OE.

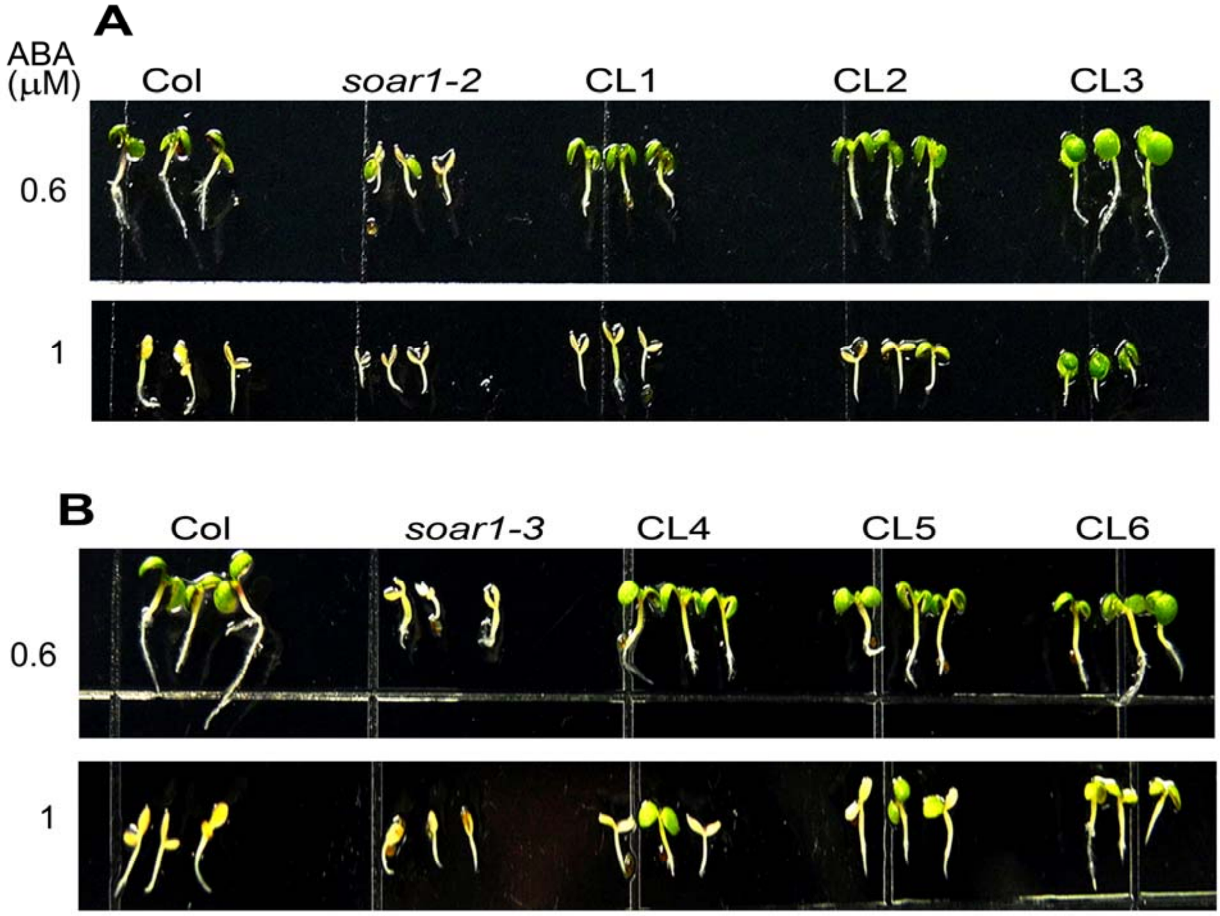

Supplementary Figure S7. Transgenic Expression of *SOAR1* Rescues the ABA-hypersensitive Phenotypes of the *soar1-2* and *soar1-3* Mutant. Seed germination and early seedling growth: seeds from the wild-type Col, *soar1-2* mutant and complementation lines (CL1, CL2 and CL3) **(A)**, and *soar1-3* mutant and complementation lines (CL5, CL5 and CL6) **(B)**, were directly planted in the MS medium supplemented with 0.6 or 1 μM (±)ABA, and the growth was investigated two weeks after stratification.

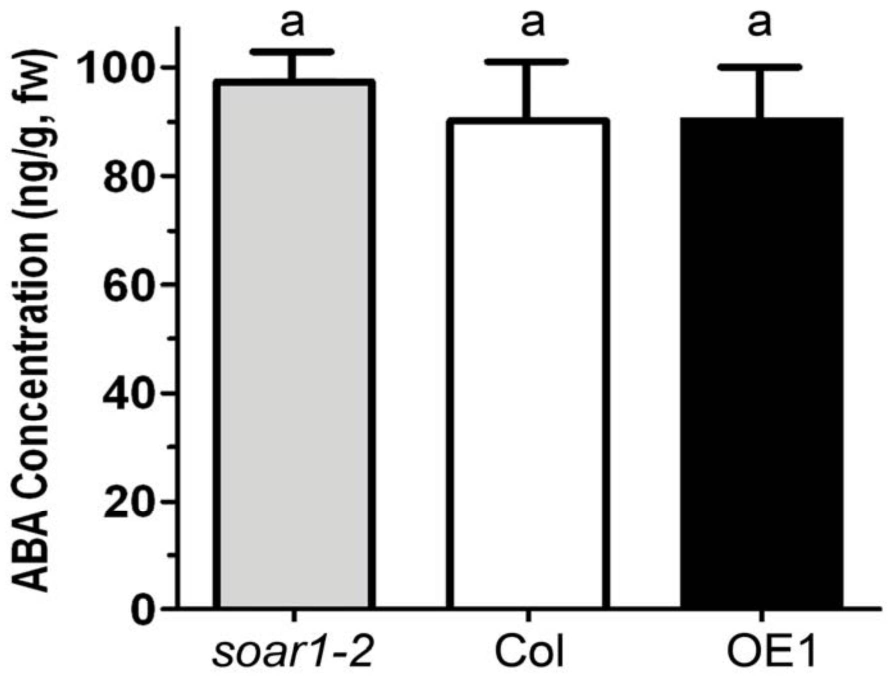

Supplementary Figure S8. ABA Concentrations in the Germinating Seeds of Different Genotypes. Each value is the mean ± SE of three biological determinations, and different letters indicate significant differences at $P < 0.05$ (Duncan's multiple-range test) when comparing the ABA concentrations among different genotypes. ABA concentration was assayed by ELISA method using a commercial immunoassay detection kit for ABA (Plant Growth Regulator Immunoassay Detection Kit, PGR1, Sigma) according to the procedures described previously (Liu R, Xu YH, Jiang SC, Lu K, Lu YF, Feng XJ, Wu Z, Liang S, Yu YT, Wang XF, Zhang DP, 2013. Light-harvesting chlorophyll *a*/*b*-binding proteins, positively involved in abscisic acid signaling, require a transcription repressor, WRKY40, to balance their function. *Journal of Experimental Botany*, 64: 5443-5456). Seeds were stratificated for 3 days at 4°C, incubated for 24 h in a light growth chamber at 20°C, collected and grinded in liquid nitrogen into fine powder, of which a sample of 0.1 g was added in to 2 mL extraction buffer containing 80% methanol and 1mM butylated hydroxytoluene (BHT) for a 4-h extraction at 4°C, then the extraction solution were centrifuged at 4℃, 12,000g. The supernatant was transferred into 1 mL extraction buffer for an extraction of 1 h. After a centrifugation at 12,000g, the supernatant was collected, frozen and dehydrated for 20 h at -60°C to remove methanol. The solute which contains ABA was collected. The sample was diluted by adding 180 µL dilution buffer into each sample in total. The dilution buffer consists of 0.2 g/L KH_2PO_4, 2.96 g/L Na_2HPO_4, and 8.5g/L NaCl. The determination of ABA concentrations in these samples was done according to the ELISA experimental procedures proposed by manufacture of the kit (Plant Growth Regulator Immunoassay Detection Kit, PGR1, Sigma).

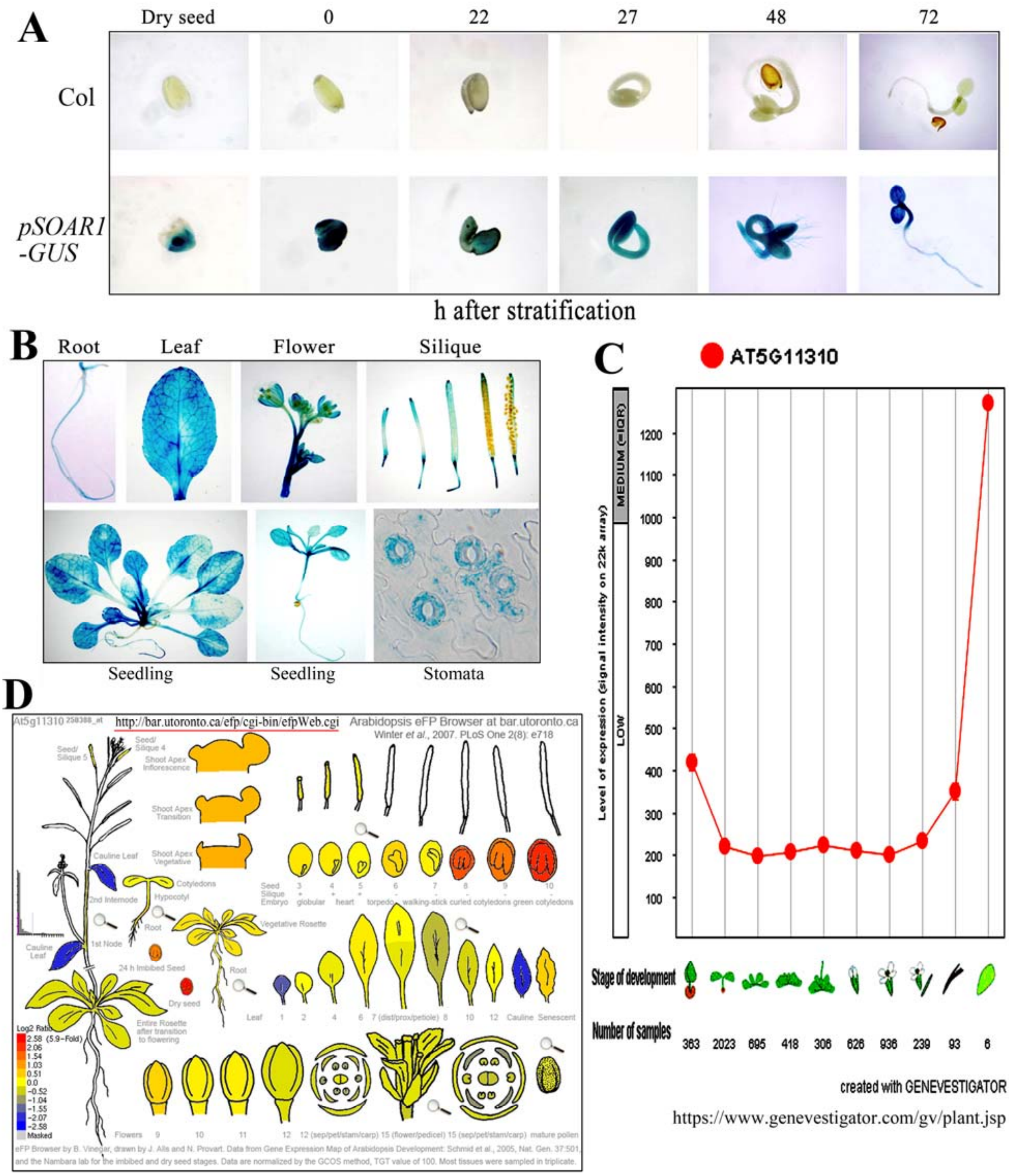

Supplementary Figure S9. *SOAR1* Is Expressed in Different Organ/Tissues with the Highest Expression Level in Seeds. GUS staining of the dry seeds and the stratified seeds (0 to 72 h after stratification) of a *SOAR1*-pomoter-*GUS* transgenic line (*pSOAR1*-*GUS*). The wild-type Col was used as a control. **(A)** GUS staining of different organ/tissues of the *SOAR1*-pomoter-*GUS* transgenic line. **(B)** The *SOAR1* expression data from a public website at http://www.genevestigator.com. **(C)** The *SOAR1* expression data from a public website at http://bar.utoronto.ca.

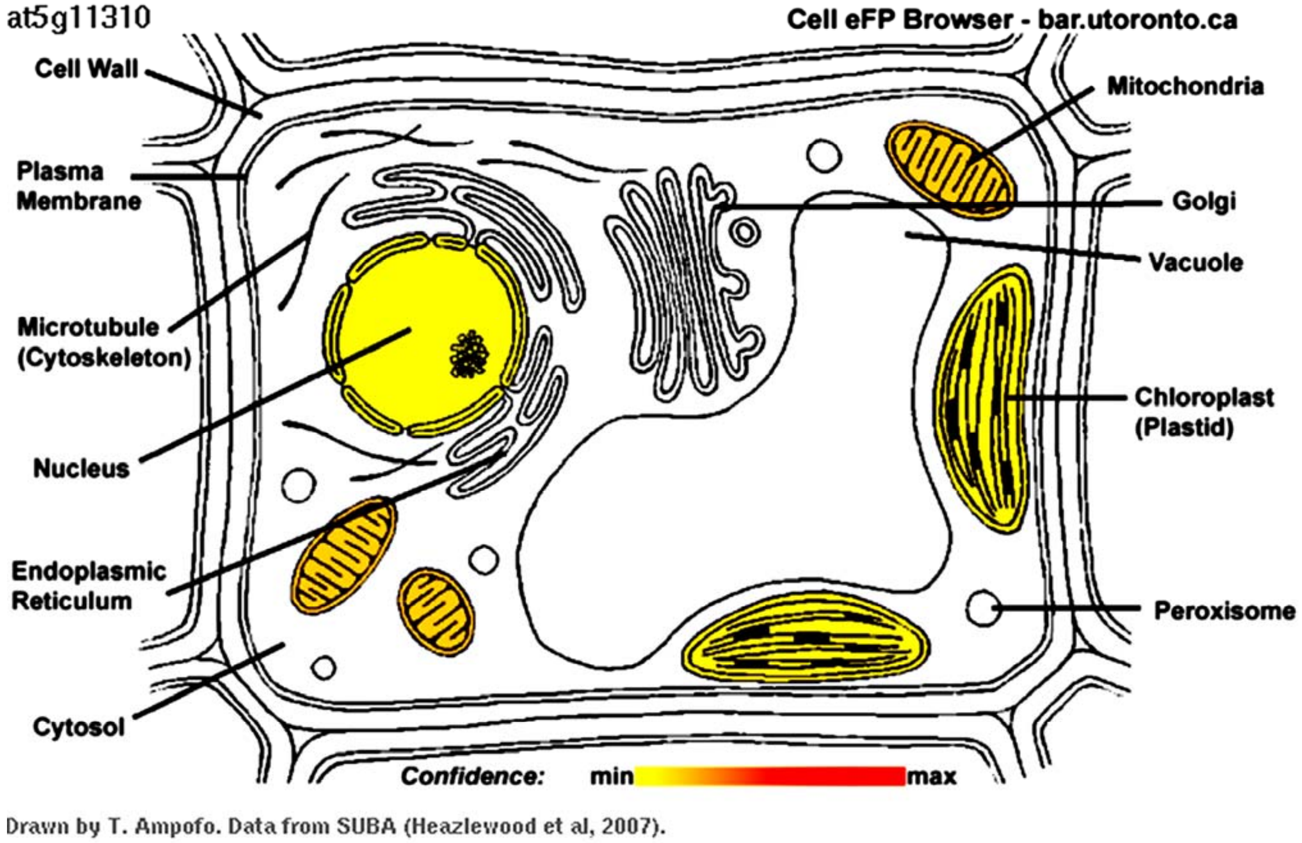

Cell eFP Browser at website: http://bar.utoronto.ca/cell_efp/cgi-bin/cell_efp.cgi

for SOAR1 (At5g11310) subcellular localization

Supplementary Figure S10. Predication of Subcellular Localization of SOAR1 Protein. The SOAR1 is predicted to localize to chloroplast, mitochondria or nucleus at the website http:/bar.utoronto.ca.

Supplementary Table S1. Primers Used in This Study.

1. Primers for quantitative real-time PCR.

Abbreviations: for, forward primer; rev, reverse primer

Actin2/8 for	5'-GGTAACATTGTGCTCAGTGGTGG-3'
Actin2/8 rev	5'-AACGACCTTAATCTTCATGCTGC-3'
SOAR1 for	5'-TACGGAACTTAGGTTGCTTGAG-3'
SOAR1 rev	5'-AACAACAGTCGGCTTCACATTC-3'
ABF4 for	5'-AACAACTTAGGAGGTGGTGGTC-3'
ABF4 rev	5'-CTTCAGGAGTTCATCCATGTTC-3'
ABI1 for	5'-AGAGTGTGCCTTTGTATGGTTTA-3'
ABI1 rev	5'-CATCCTCTCTCTACAATAGTTCGCT-3'
ABI2 for	5'-GATGGAAGATTCTGTCTCAACGATT-3'
ABI2 rev	5'-GTTTCTCCTTCACTATCTCCTCCG-3'
ABI3 for	5'-ACCCAAATCGGAGAAACCTGTG-3'
ABI3 rev	5'-GAGGTTCCGATGTCTTCCATGG-3'
ABI4 for	5'-GGGCAGGAACAAGGAGGAAGTG-3'
ABI4 rev,	5'-ACGGCGGTGGATGAGTTATTGAT-3'
ABI5 for	5'-CAATAAGAGAGGGATAGCGAACGAG-3'
ABI5 rev	5'-CGTCCATTGCTGTCTCCTCCA-3'
DREB1A for	5'-GATCAGCCTGTCTCAATTTC-3'
DREB1A rev	5'-CTTCTGCCATATTAGCCAAC-3'
DREB2A for	5'-AAGGTAAAGGAGGACCAGAG-3'
DREB2A rev	5'-ACACAACCAGGAGTCTCAAC-3'
MYB2 for	5'-TGCTCGTTGGAACCACATCG-3'
MYB2 rev	5'-ACCACCTATTGCCCCAAAGAGA-3'
RAB18 for	5'-CAGCAGCAGTATGACGAGTA-3'
RAB18 rev	5'-CAGTTCCAAAGCCTTCAGTC-3'
PYR1 for	5'-GAACAAAACTTCGAGATGCG-3'
PYR1 rev	5'-GGAGTTACGAGCCATAGCTTC-3'
PYL2 for	5'-ATGAAGAGCAGAAAACCCTC-3'
PYL2 rev	5'-TTCAAGAACTCATTGACCGA-3' -3'
PYL4 for	5'-GCGGCTGAGAGCAAGAAGAA-3'
PYL4 rev	5'-GTGAAAGCCGGAATAGGGCT-3'
PYL9 for	5'-ACGTACGGACGCATCATCAA-3'
PYL9 rev	5'-ACGGTTTGTATTTCTGCGGC-3'
PYL7 for	5'-TCCTCAAGGCAACACCAAAGA-3'
PYL7 rev	5'-AAGCGTTACAGAAAGTTGCGA-3'
RD29A for	5'-ATCACTTGGCTCCACTGTTGTTC-3'
RD29A rev	5'-ACAAAACACACATAAACATCCAAAGT-3'
RD29B for	5'-CTTGGCACCACCGTTGGGACTA-3'
RD29B rev	5'-TCAGTTCCCA GAATCTTGAACT-3'
SnRK2.2 for	5'-ATATGCCATCGGGATCTGAA-3'
SnRK2.2 rev	5'-TTGGTTGGGAATGAAGAACAG-3'
SnRK2.3 for	5'-GTTGGATGGAAGTCCTGCTC-3'
SnRK2.3 rev	5'-TGCCATCATATTCCTGACGA-3'

2. Primers for identification of the mutants

(1) *soar1-2* (stock number: FLAG_546D07)

LB4: 5'-CGTGTGCCAGGTGCCCACGGAATAGT-3' (Left border primer, LB)

soar1-2 LP: 5'-GTGAACCAACTCAACACTCGG-3' (Left primer, LP)

soar1-2 RP: 5’-TCACCGCAATGTATCTACCATC-3’ (Right primer, RP)

(2) *soar1-3* (stock number: FLAG_500B04)
LB4: 5’-CGTGTGCCAGGTGCCCACGGAATAGT-3’
soar1-3 LP: 5’-GCTCGTATAGCTTGTTGCACC-3’
soar1-3 RP: 5’-ATAACCACATCCATTGCCTTG-3’
(3) *abi1-3* (stock number: SALK_076309)
LBa1: 5’-TGGTTCACGTAGTGGGCCATCG-3’
abi1-3 LP: 5’-TCAGGAATGATGGATGGTTTC-3’
abi1-3 RP: 5’-GGTTTCGTTACCGGAGACTTC-3’
(4) *abi2-2* (stock number: SALK_015166)
LBa1: 5’-TGGTTCACGTAGTGGGCCATCG-3’
abi2-2 LP: 5’-AAACTGTTGGGTCTACCTCGG-3’
abi2-2 RP: 5’-ACCATCCCATATTCTGGTTGG-3’
(5) *abi5-1* (stock number: CS8105)
abi5-1 LP: 5’-ACAGCTTTATGGTGTGTTTCAA-3’
abi5-1 RP: 5’-CCATCTGAAGACACCGGGCTTAA-3’CGGGC
PCR products of heterozygotes or wild-type could be digested by the *Hae*III enzyme, while those of the homozygotes could not be digested.

3. Primers for cloning full-length *SOAR1*
Forward: 5’-ATGAACTCTCTGTTCACCGCC-3’
Reverse: 5’-TCACTCAAAATCCCCTGCATCTCC-3’
For the construct *CaMV 35S-SOAR1* (used to create transgenic *SOAR1*-overexpression lines)
Forward: 5’-GCTCTAGAATGAACTCTCTGTTCACCGCC-3’
Reverse: 5’-GGGGTACCCTCAAAATCCCCTGCATCTCC-3’

4. Primers for cloning native promoter of *SOAR1*
Forward: 5’-AACTGCAGAAATATGTGTAGGTGGGGGAGAG-3’
Reverse: 5’-GCTCTAGATTTCTGCCGCCGTTTATTAGAA-3’

5. Primers for identification of the *soar1-1* dominant mutant by tail-PCR
(1) Long specific primers (SP) for the vector:
SP1, 5’-TACTCGCCGATAGTGGAAACCG-3’
SP2, 5’-AAAGAAATAGAGTAGATGCCGACCG-3’
SP3, 5’-TACTCGCCGATAGTGGAAACCG-3’
(2) Short arbitrary degenerate primer (AD) for the genome:
AD1, 5’-NTCGASTWTSGWGTT-3’
AD2, 5’-NGTCGASWGANAWGAA-3’
AD3, 5’-WGTGNAGWANCANAGA-3’
AD4, 5’-AGWGNAGWANCAWAGG-3’
(W = A or T; S = C or G; N = A, C, G or T).

6. Primers for protoplast and onion epidermis transformation
(1) Primers for cloning the full-length *SOAR1*:
Forward, 5’-CCTTAATTAAATGAACTCTCTGTTCACCGCC-3’
Reverse, 5’-AGGCGCGCCACTCAAAATCCCCTGCATCTCC-3’

(2) Primers for cloning the SOAR1 fragment from 106 to1809 bp downstream from transcription start site:
Forward, 5'-CCTTAATTAAATGCCGCTCATTCCCGTCGA-3'
Reverse, 5'-AGGCGCGCCACTCAAAATCCCCTGCATCTCC-3'
(3) Primers for cloning *PYR1*:
Forward, 5'-CCTTAATTAAATGCCTTCGGAGTTAACACC-3'
Reverse, 5'-AGGCGCGCCACGTCACCTGAGAACCACTTC-3'
(4) Primers for cloning *FBI1*:
Forward: 5'-CCTTAATTAAATGTCGAATAATCAAGCTTT-3'
Reverse: 5'-AGGCGCGCCATAGTCTTCTCATCGCATGGG-3'

7. Primers for semiquantitative RT-PCR for *SOAR1*

SOAR1 forward: 5'-ATGAACTCTCTGTTCACCGCCT-3'

SOAR1 reverse: 5'-TCACTCAAAATCCCCTGCATCT-3'

Actin2/8 forward: 5'-GGTAACATTGTGCTCAGTGGTGG-3'

Actin2/8 reverse: 5'-AACGACCTTAATCTTCATGCTGC-3'

8. Primers for production of the anti-SOAR1 (299-602 aa) antibody

Forward: 5'-CGTCGTGTTCAGATTGCTATGGA-3'

Reverse: 5'-TCACTCAAAATCCCCTGCATCT-3'
